# Concept and feasibility of privacy-preserving record linkage of cancer registry data and claims data in Germany: results from the DigiNet study on stage IV non-small cell lung cancer

**DOI:** 10.1007/s00432-025-06384-7

**Published:** 2025-12-04

**Authors:** Anika Kästner, Christopher Hampf, Pia Naumann, Lizon Fiedler-Lacombe, Anna Kron, Anna Spier, Dusan Simic, Leonie Eilers, Aleksandra Graw, Sebastian Bartholomäus, Andreas Stang, Daniela Reil, Renate Kirschner-Schwabe, Jessica Isabel Selig, Jörg Wulff, Patrik Dröge, Thomas Ruhnke, Christian Günster, Uwe Nußbaum, Ursula Marschall, Juliane Mohnke, Anja Hebbelmann, Uwe Lührig, Anna Rasokat, Vanessa Mildenberger, Stephanie Stock, Florian Kron, Jürgen Wolf, Martin Bialke, Dana Stahl, Neeltje van den Berg, Wolfgang Hoffmann

**Affiliations:** 1 Institute for Community Medicine, Section Epidemiology of Health Care and Community Health, University Medicine Greifswald, Ellernholzstraße 1-2, 17487 Greifswald, Germany; 2 University Medicine Greifswald - Independent Trusted Third Party, Greifswald, Germany; 3 National Network Genomic Medicine Lung Cancer, University Hospital of Cologne, Cologne, Germany; 4 Department I of Internal Medicine, Center for Integrated Oncology Aachen Bonn Cologne Duesseldorf, Lung Cancer Group Cologne, University Hospital of Cologne, Cologne, Germany; 5 Institute of Health Economics and Clinical Epidemiology, Faculty of Medicine, University of Cologne, University Hospital of Cologne, Cologne, Germany; 6 FOM University of Applied Sciences, Essen, Germany; 7 Cancer Registry North-Rhine Westphalia, Bochum, Germany; 8 Clinical-Epidemiological Cancer Registry Brandenburg-Berlin, Cottbus, Germany; 9 Cancer Registry Saxony, Dresden, Germany; 10 AOK Research Institute, Berlin, Germany; 11 BARMER, Wuppertal, Germany; 12 IKK classic, Dresden, Germany; 13 Mobil Krankenkasse, Hamburg, Germany

**Keywords:** Record linkage, Exact linkage, Cancer registry, Health insurance, Claims data

## Abstract

**Objective:**

While cancer registry and health insurance data are valuable resources for oncological health services research, these are rarely linked at the individual level due to data protection concerns and technical limitations. The prospective, controlled cohort study *DigiNet* aims to optimize personalized care for patients with stage IV non-small cell lung cancer (NSCLC) in the German study regions Berlin, Saxony and North Rhine-Westphalia. The population-based control group (pCG) was identified through cohort matching within the participating cancer registries. For health economic analyses, case-specific linkage of cancer registry data with claims data without informed consent was required.

**Methods:**

A privacy-preserving record linkage (PPRL) concept was developed, ensuring that no conclusions about individual identities can be drawn. The approach relied on irreversible encryption of the statutory health insurance number (KVNR) within the data-holding institutions, using a study-specific configuration of a publicly available software.

**Results:**

Following cohort matching in the cancer registries, *N* = 9,597 pCG cases with stage IV NSCLC diagnosis between June 2022 and March 2024 were identified. Of these, *n* = 1,437 (15.0%) had insurance coverage with one of three participating statutory health insurance funds and were eligible for PPRL. Among those, 94.2% (*N* = 1,354) were successfully linked with claims data. A trusted third party performed the linkage based on encrypted identifiers, removed the linkage keys, and provided the data to the evaluating parties.

**Conclusions:**

This study demonstrates the feasibility of PPRL of cancer registry and claims data in a real-world oncological research setting. The concept is transferable to other research contexts requiring secure, identifier-based linkage without disclosure of personal identifiers.

**Supplementary Information:**

The online version contains supplementary material available at 10.1007/s00432-025-06384-7.

## Purpose

With a total of 56,690 newly diagnosed cases and 44,817 deaths in 2020, lung cancer is the third most prevalent cancer and the most frequent cause of cancer death in Germany (“Association of Epidemiological Cancer Registries in Germany e. V. (GEKID); German Centre for Cancer Registry Data (ZfKD) at the Robert Koch Institute. Cancer in Germany 2019/2020,” 2023). Notably, 57% of patients with lung cancer are first diagnosed at metastatic stage IV, with a 1-year survival probability of less than 35% (Emrich and Kraywinkel 2022). Over the last few decades, however, systemic cancer therapy concepts have substantially evolved from a previously uniform chemotherapy approach to a more personalized therapy concept, significantly improving both prognosis and quality of life (QoL) (Schuler, 2023). About 30% of NSCLC patients in Germany harbor targetable genetic alterations (Frost et al. 2022), and the subsequent administration of targeted therapies can significantly improve the prognosis and the QoL of these NSCLC patients (Barlesi et al. 2016; Kästner et al. 2024; Kris et al. 2014; Ramalingam et al. 2020). However, implementing personalized care remains challenging due to rapid advancements in the field, an expanding spectrum of actionable mutations, and the increasing complexity of treatment decisions. As one consequence, still not all advanced NSCLC patients in Germany receive molecular testing for all relevant targetable driver mutations (Griesinger et al. 2021).

Therefore, the national Network Genomic Medicine (nNGM) Lung Cancer aims to ensure that all patients with NSCLC receive comprehensive genetic testing and cancer treatment based on the latest scientific advancements (Kästner et al. 2024). The network’s concept is designed to ensure that patients receive cancer care close to home through numerous outpatient and inpatient oncologists (*nNGM* network partners), who collaborate closely with specialized oncological centers (*nNGM* centers). At these centers, comprehensive standardized molecular diagnostics are performed to identify all known therapeutically relevant driver mutations. Additionally, harmonized therapy information including details on clinical trials, in-label and off-label medication as well as counseling, are provided to guide complex treatment decisions in an interdisciplinary manner. Today, the *nNGM* includes 29 *nNGM* centers, and nearly 500 affiliated *nNGM* partners across the inpatient and outpatient care sector. A detailed description of the *nNGM* can be found in other sources (Büttner et al. 2019; Kästner et al. 2024).

The *DigiNet* study builds upon the foundational structures of the *nNGM* in the model regions East (German federal states of Berlin and Saxony) and West (North Rhine-Westphalia) and aims to digitally strengthen the collaboration between the *nNGM* centers and practitioners in routine care to further optimize the personalized treatment of patients with stage IV NSCLC (Kästner et al. 2025). The *DigiNet* intervention group is actively recruited in the model regions as single-arm cohort based on written informed consent. To evaluate the benefits of the *DigiNet* intervention, a population-based control group (pCG) is subsequently identified through cohort matching within the federal state cancer registries of the model regions. The cancer registries therefore serve both to define the pCG in *DigiNet* and to provide comprehensive clinical and tumor-related data for comparison with the intervention group. Further details of the *DigiNet* study can be found elsewhere (Kästner et al. 2025).

In addition to the evaluation of clinical outcomes such as overall survival (OS) and time on first-line treatment as part of the *DigiNet* study, a health economic evaluation is performed. For this purpose, data from cancer registries of the pCG and claims data from statutory health insurance providers must be linked on a case-specific basis to ensure a robust and valid analysis. As no study-specific declaration of informed consent is available from the pCG, a privacy-preserving record linkage (PPRL) must be implemented.

## Methods

The *DigiNet* study is funded by the Innovation Fund of the Federal Joint Committee (Gemeinsamer Bundesausschuss, G-BA) in Germany (01NVF20021). The Ethics Committee of the University Hospital of Cologne reviewed and approved the *DigiNet* study protocol (21-1521). The study was first registered retrospectively on ClinicalTrials.gov on December 12, 2022 (NCT05818449).

### Brief overview of the study design, timeline and eligibility criteria of DigiNet

*DigiNet* is a prospective, controlled, non-randomized, non-blinded multicenter cohort study, conducted in the two model regions East (German federal states of Berlin and Saxony) and West (North Rhine-Westphalia). The study has a total duration of four years (10/2021–09/2025), comprising a 22-month recruitment phase and a 12-month follow-up phase. Patients who are newly diagnosed with stage IV NSCLC between June 1, 2022, and March 31, 2024, and have received comprehensive molecular diagnostics at the participating *nNGM* centers of the model regions are eligible for inclusion in the intervention group. All participating *DigiNet* partners are listed on clinicaltrials.gov (*ClinicalTrials.gov: Improvement of Personalized Lung Cancer Care Through Digital Connection and Patient Participation (DigiNet): NCT05818449*, 2024) and all participating *nNGM* partners can be found on the *nNGM* website (*Arztsuche nNGM Netzwerkpartner finden*, 2025). The pCG comprises patients newly diagnosed with stage IV NSCLC in the same period and regions who did not participate in *nNGM* and, consequently, were not enrolled in *DigiNet*.

### Data sources (cancer registries and statutory health insurances) and legal basis

The Cancer Registry North Rhine-Westphalia (NRW), the Cancer Registry Saxony, and the Clinical-Epidemiological Cancer Registry Brandenburg-Berlin (KKRBB) participate in *DigiNet* and provide data for the pCG following the approval of a data access request, in compliance with the current use and access regulations. The legal framework for utilizing cancer registry data for research purposes is based on the respective cancer registry laws of the federal states.

As part of *DigiNet*, data are requested in a standardized format (*onkologischer Basisdatensatz*, oBDS (*Bundeseinheitlicher Onkologischer Basisdatensatz*, 2025)) and include sociodemographic variables such as age, sex, general health status (ECOG-PS), and history of previous malignancies. Additionally, information on NSCLC diagnosis is obtained, including the date of initial diagnosis, ICD-O-3 code (covering morphology and topography (Fritz et al. 2000)), tumor grading, clinical and pathological TNM classification, as well as the presence and localization of metastases. Furthermore, therapy-related data, including details on residual tumor status, radiotherapy, and systemic therapies, are requested, along with patient outcome data such as tumor response status over the course of the disease and the date and cause of death.

As part of routine cancer registration, each cancer registry also documents personal identifying information (PII) such as first name, last name, date of birth, postal address, statutory health insurance provider, and the health insurance number (*Krankenversichertennummer*, KVNR). These PII are collected for administrative purposes, including the linkage of follow-up reports and billing purposes. Beyond this administrative use, they may also be utilized for research purposes to enable case-specific data linkage under strict legal and data protection conditions as specified in the respective cancer registry laws.

In addition to the data from the state cancer registries, data from the statutory health insurance providers participating in the *DigiNet* study (*AOK Rheinland/Hamburg*, *AOK NordWest*, *AOK PLUS*, *AOK Nordost*, *BARMER*, *IKK classic*, and *Mobil Krankenkasse*) are necessary for the health economic evaluation. The dataset includes demographic information, insurance periods, outpatient and inpatient treatment data, medication, medical aids and remedies, care requirements, and time periods of incapacity for work as well as the associated statutory health insurance expenditures. The legal basis for utilizing claims data for research purposes is § 75 of the Tenth Book of the German Social Code (SGB X), which governs the transfer of claims data for research and planning. The provision of these data requires approval following an application submitted to the German Federal Office for Social Security (Bundesamt für Soziale Sicherung) and, regarding regional health insurance funds, from the respective state-level supervisory bodies.

Since tumor stage, diagnosis date, and histological type (morphology) are not recorded at the German statutory health insurance providers, selecting stage IV NSCLC patients for the pCG based solely on claims data carries a high risk of selection bias. To ensure a robust and valid analysis, it is therefore essential to link data from cancer registries and health insurance providers on a case-specific basis.

The data use and linkage applications for the pseudonymized data provision of the pCG by the Cancer Registry NRW, the Cancer Registry Saxony, and the KKRBB were reviewed by independent advisory boards of the cancer registries and were fully approved. Additionally, the claims data use and linkage application was thoroughly reviewed for accordance with § 75 SGB X and fully approved by the German Federal Office for Social Security and the state-level supervisory bodies.

In the following, we outline the PPRL procedure used to link cancer registry data with claims data from *BARMER*, *IKK classic*, and *Mobil Krankenkasse*, covering around 16.2% of the German statutory health-insured population (data as of July 2025 (Krankenkassen.Deutschland 2025)). The linkage of cancer registry and AOK data provided by the Scientific Institute of the AOK (Wissenschaftliches Institut der AOK, WIdO) followed an institute-specific approach, which is briefly described in the next section.

### Requirements of the record linkage process

In principle, record linkage can be either deterministic or probabilistic. Deterministic linkage compares predefined variables such as name, date of birth, or gender for exact or rule-based agreement, whereas probabilistic linkage calculates the probability that records from different sources refer to the same individual based on the similarity of multiple variables (Intemann et al. 2023; March et al. 2018). Each approach has specific advantages and limitations. Probabilistic linkage allows for error tolerance in the identifiers used, however, depending on the threshold used to categorize probability values as either match or non-match, different proportions of false positives (homonym error rate) and false negatives (synonym error rate) may occur (Brenner et al. 1997; March et al. 2018). Deterministic or exact linkage, in contrast, ensures a high degree of linkage accuracy when based on a unique identifier, as even the slightest deviations in this identifier preclude a match.

Therefore, in the *DigiNet* study, a deterministic linkage approach was implemented using the KVNR as a unique identifier to ensure exact, case-specific linkage. The KVNR is a standardized identifier in Germany, which consists of ten characters, comprising one letter followed by nine digits. Due to this structured format, even minor variations in a single character (for example, an increase or decrease of one digit) can result in KVNRs that appear highly similar but correspond to different individuals. Therefore, an exact linkage prevents wrong linkages between persons with similar KVNRs. The correctness of the KVNR must be ensured by the data-holding institutions. Errors like transposed digits can be detected by cancer registries by checking the check digit within the KVNR. The check digit is computed from the first nine digits of the KVNR according to a defined rule to detect input errors (GKV-Spitzenverband).

This KVNR-based deterministic linkage approach was applied for the participating statutory health insurance providers *BARMER*, *IKK classic*, and *Mobil Krankenkasse*. As the WIdO exclusively stores pseudonymized claims data of the AOK health insurance funds and has no access to PII such as the KVNR, the record linkage concept described in the following sections was not applicable. Instead, the cancer registries used the same identifier (KVNR) but applied a WIdO-specific encryption algorithm to generate pseudonyms identical to those available in the WIdO for deterministic record linkage. As this institute-specific encryption algorithm is restricted to the WIdO and is not publicly available, no further details are provided.

As no study-specific declaration of consent is available for the pCG, a PPRL procedure must be implemented to prevent any identification of individuals within the pCG. The term PPRL refers to record linkage procedures that link data records from different data sources without exchanging PII (Intemann et al. 2023). Generally, encrypted identifiers (e.g., control numbers, or Bloom filters (Bloom 1970) are utilized for this purpose, allowing no conclusions to be drawn about the identity of the persons involved, or only with disproportionate effort.

The PPRL process must adhere to the following requirements and principles:

1. **Confidentiality of identifying data**: PII of the pCG must never leave the data-holding institutions (participating cancer registries and statutory health insurance providers).
2. **Exact linkage**: The encryption algorithm must ensure that based on a uniquely identifying identifier, the same encryption key is always generated, guaranteeing exact (case-specific) linkage of records from different data sources.
3. **Secure encryption**: The uniquely identifying identifier must be irreversibly encrypted using a secure procedure.
4. **Restricted encryption knowledge**: The encryption algorithm must remain confidential and must not be disclosed to anyone outside the data-holding institutions.
5. **Record linkage and pseudonymization by an independent trusted third party**: An independent trusted third party (TTP) must be responsible for linking the data sources based on the encrypted identifiers (linkage keys) (Bialke et al. 2015). The TTP must remove the linkage key and generate new pseudonyms before passing on the pseudonymized, merged dataset to the evaluating bodies.
6. **Irreversible deletion**: The mapping (i.e., the combination and assignment) of linkage keys to pseudonyms must be irreversibly deleted by the TTP immediately after the linkage process is completed and the objectives of the evaluation have been achieved.

To enable the technical implementation of this process, the data-holding institutions must ensure that the required rights are granted to install the external software in their respective systems.

Moreover, all data is transferred using Cryptshare (Pointsharp GmbH, version 7.1.0), whose HTTPS-secured web interface enables the encrypted and password-protected provision of data via a server operated by the University Medicine Greifswald.

## Results

The independent TTP of the University Medicine Greifswald was responsible for linking the data from the federal state cancer registries and health insurance providers. Therefore, the TTP was divided into two technically, organizationally, and personnel-wise separate units: The unit “TTP Greifswald” exclusively manages PII, whereas the unit “Data Merging” exclusively manages anonymized or pseudonymized medical data (e.g. pseudonymized data from the NDP).

### Selection of eligible cases in the cancer registries

After recruitment and follow-up of the *DigiNet* intervention group had been completed at the end of March 2025, the selection of eligible cases of the pCG was conducted within the participating cancer registries via cohort matching.

**Fig. 1:**
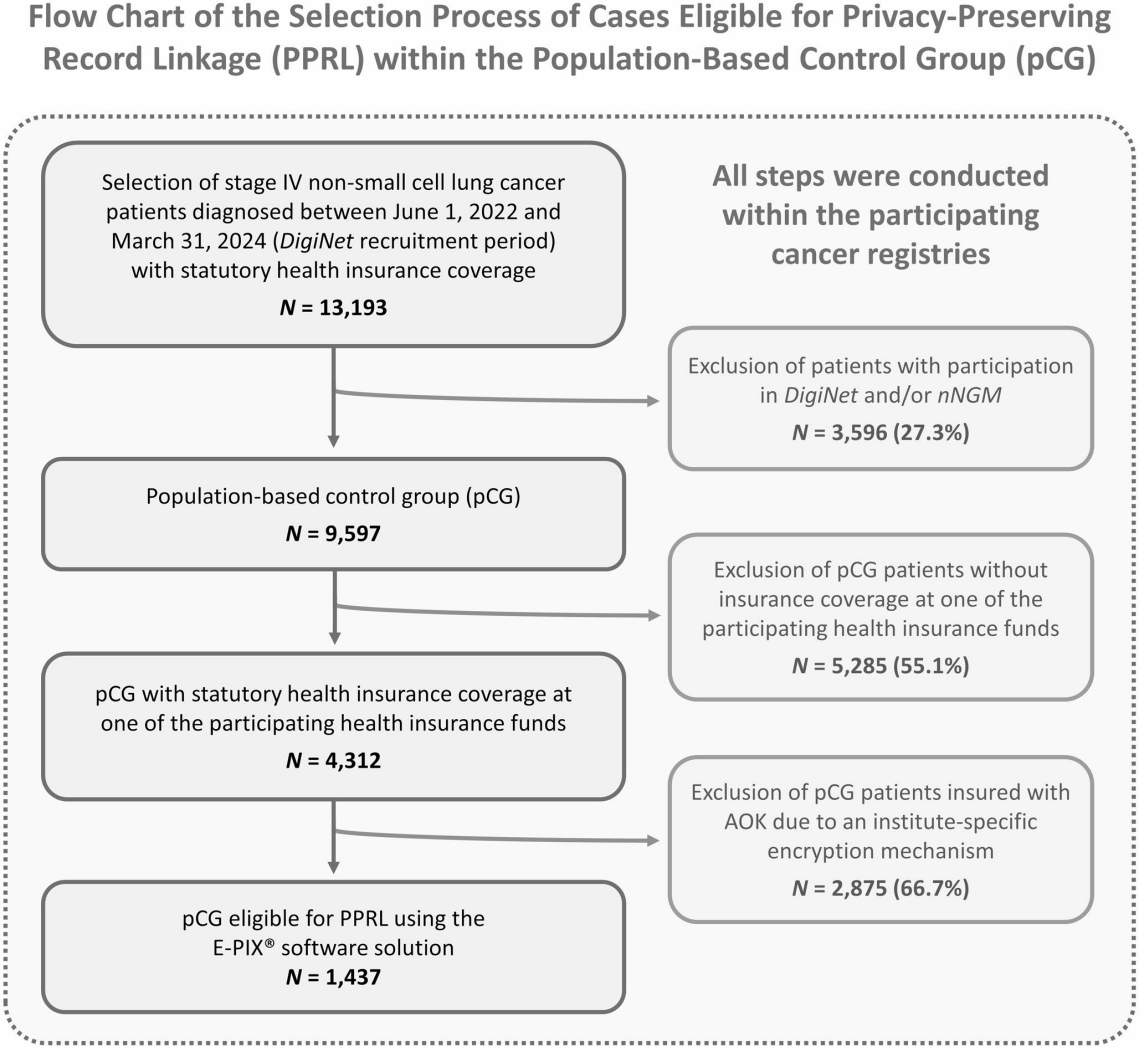
Flow chart of the selection process of cases eligible for privacy-preserving record linkage (PPRL) within the population-based control group (pCG)

For this purpose, the cancer registries first selected patients diagnosed with stage IV NSCLC in the recruitment period based on their records (target population, see Fig. 1). This resulted in *N* = 13,193 stage IV NSCLC patients with diagnosis between June 1, 2022, and March 31, 2024 (DigiNet recruitment period), who were covered by statutory health insurance. Then, the KVNRs of the *DigiNet* intervention group and the *nNGM* control group were sent to the respective cancer registries via the TTP unit “TTP Greifswald”. An informed written declaration of consent was available for the transfer of the KVNR to the cancer registries. Based on the KVNR, the cancer registries excluded *DigiNet* and/or *nNGM* cases from the target population, leaving *N* = 9,597 lung cancer cases that had not participated in either *DigiNet* or *nNGM* and thus comprise the pCG (see Fig. 1). Among these pCG cases, health insurance information recorded within the cancer registries indicated that *n* = 1,437 (15.0%) patients were insured with one of the participating statutory health insurance providers. Only these registry-selected cases were eligible for PPRL, enabling case-specific linkage of cancer registry and corresponding claims data (see Fig. 1).

### Phase 1 of the PPRL workflow: generation and matching of linkage keys

**Fig. 2:**
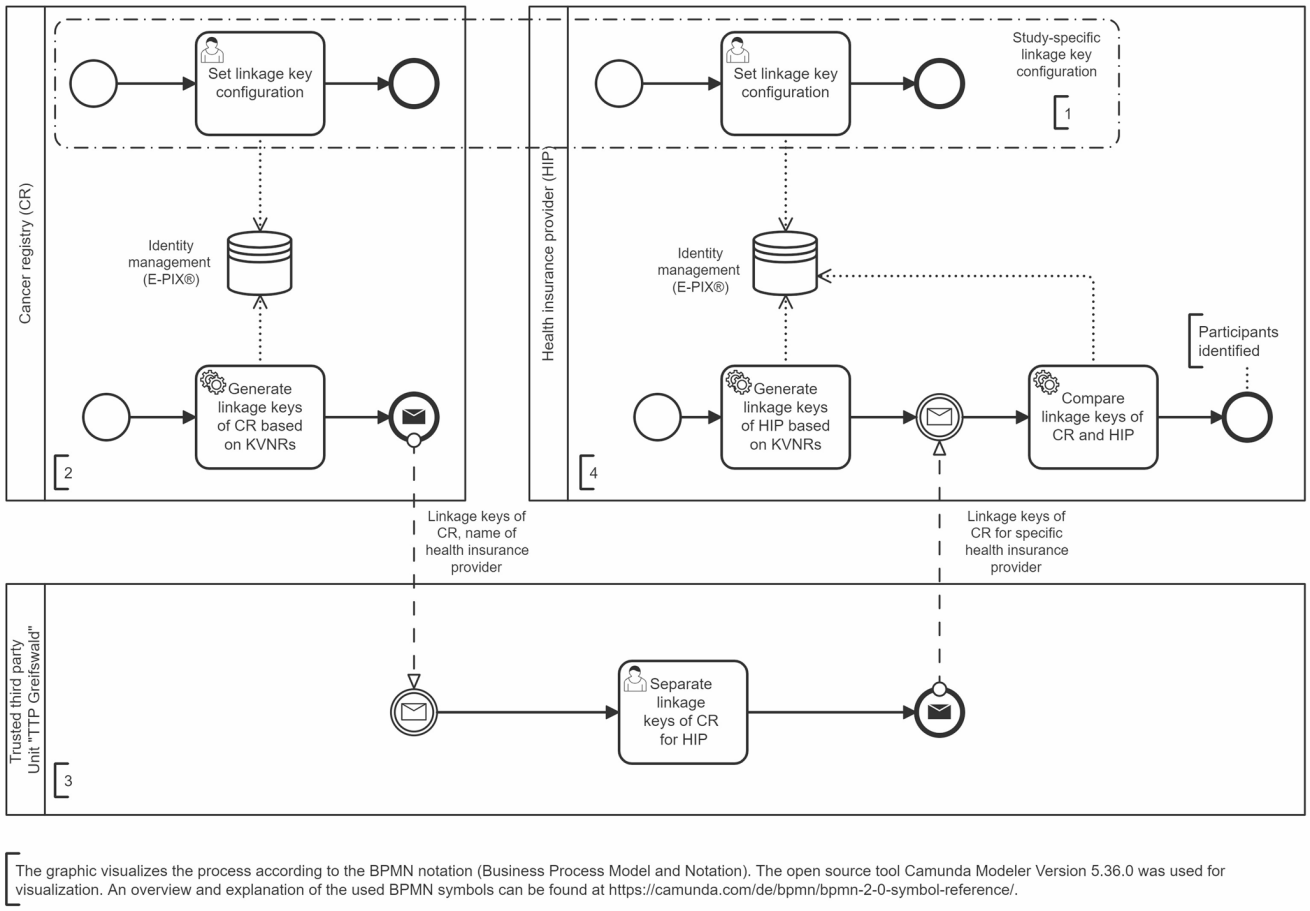
Business process model and notation (BPMN) diagram of the privacy-preserving record linkage (PPRL) process implementing the study-specific configuration using the E-PIX^®^ software solution (phase 1)

To enable PPRL, linkage keys were generated separately within each data-holding institution, based exclusively on the health insurance number (KVNR). The overall process is visualized in Fig. 2.

First, within each participating cancer registry, the KVNRs were irreversibly encrypted using the E-PIX^®^ software solution with a study-specific configuration (see Fig. 2, step 1 and 2). The implementation of this study-specific configuration using the E-PIX^®^ is explained in detail in the section ‘Implementing the E-PIX^®^ software solution’. The generated linkage keys, together with the name of the patient’s health insurance provider, were then sent to the “TTP Greifswald” unit.

The unit “TTP Greifswald” then transferred the linkage keys to the respective health insurance providers participating in *DigiNet* (see Fig. 2, step 3). There, similar to the cancer registries, the health insurance providers first selected cases with inpatient or outpatient lung cancer diagnosis (ICD-10: C34.-) within the recruitment period in the three federal states of the model regions. These selection criteria were applied solely to narrow down the list of KVNRs to be encrypted for generating the linkage keys, not to define the study cohort.

Using the E-PIX^®^ with the study-specific configuration as applied in the cancer registries (see Fig. 2, step 1), the selected KVNR were then irreversibly encrypted within the participating health insurance providers (see Fig. 2, step 4). The resulting linkage keys were then compared with those received from the cancer registries via the TTP to detect matching linkage keys.

### Phase 2 of the PPRL workflow: data transfer and record linkage

**Fig. 3:**
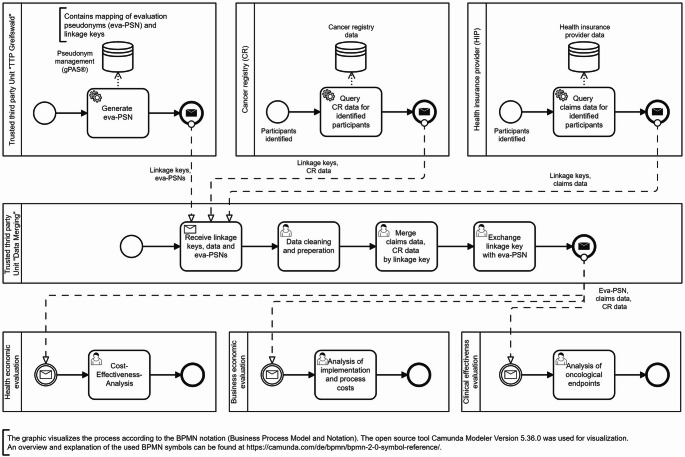
Business Process Model and Notation (BPMN) diagram of the privacy-preserving record linkage (PPRL) process, showing the data flows between data-holding institutions, the trusted third party, and evaluating parties (phase 2)

After matching linkage keys had been detected, the corresponding claims data with the linkage keys were transferred to the “Data Merging” unit of the independent TTP (see Fig. 3). In parallel, the cancer registries also transferred their data of the pCG cases along with the linkage keys to the “Data Merging” unit. If no linkage key was available, as the health insurance provider does not participate in *DigiNet*, the cancer registry data was provided in an anonymized format to the “Data Merging” unit.

Case-specific linkage of cancer registry and health insurance data was then performed based on the linkage key by the unit “Data Merging” of the independent TTP (see Fig. 3).

After completion of the linkage process, the linkage keys were removed, and replaced by evaluation pseudonyms (eva-PSN) generated with the software gPAS^®^ by the TTP. With the eva-PSN, the pCG cancer registry and claims data were finally provided to the evaluating parties by the TTP unit “Data Merging”. The mapping (i.e., the combination and assignment) between the linkage keys and evaluation pseudonyms were irreversibly deleted by the TTP immediately after the linkage process was completed and the objectives of the evaluation have been achieved.

### Implementing the E-PIX^®^ software solution: *DigiNet* configuration

This linkage uses the publicly available E-PIX^®^ (Enterprise Identifier Cross-Referencing) software, an identity management solution designed to manage and compare PII for record linkage purposes (Hampf et al. 2020). E-PIX^®^ supports deterministic and probabilistic record linkage methods to link datasets from different data sources that hold information on the same person.

In settings where data protection requirements prohibit the transfer of PII to a TTP, the E-PIX^®^ enables PPRL. Therefore, record linkage is performed using Bloom filters, which are bit vectors that encode data into binary patterns, allowing encrypted comparison without revealing the original identifiers (Bloom 1970). In contrast to other hashing methods, Bloom filters enable comparisons for similarity. These Bloom filters can then be used as linkage keys within the PPRL framework.

For this purpose, local E-PIX^®^ instances were installed at all participating data-holding institutions. Detailed instructions for installation of the local E-PIX^®^ instance are provided in the E-PIX^®^ installation and configuration guideline (see Supplementary File 1, Chap. 2).

The linkage based on the publicly available E-PIX^®^ ensures through technical and organizational measures that no conclusions about the identities of individuals can be drawn. Therefore, the configuration of *DigiNet* is based on random hashing (Schnell and Borgs 2016). Thereby, the KVNR is divided into smaller substrings, often of the length of two, so called bigrams. The common name is n-gram. In contrast to other generating methods, random hashing uses a lookup table for all expected n-grams with the pre-defined number of bit positions for the specific n-gram. To calculate these bit positions across all involved parties in the same manner, a pseudo random number generator (PRNG) is initialized with a pre-defined seed. A PRNG generates the same sequence of random numbers when the same seed is used. After encoding the KVNR into a random hashed Bloom filter, a balanced Bloom filter is generated, which is a hardening technique that prevents reidentification of individuals without additional information (Schnell and Borgs 2016). For this purpose, an inverted copy (zeros become ones and ones become zeros) is attached to the given Bloom filter. The bit positions are then randomly scrambled using a second PRNG initialized with a second seed. This method guarantees, that the resulting Bloom filter has the same number of ones and zeros. This doubles the number of bits constituting the Bloom filter. Step-by-step instructions for configuring the E-PIX^®^ are provided in Chap. 3 of the E-PIX^®^ installation and configuration guideline (see Supplementary File 1).

To protect the privacy of individuals within the pCG, stakeholders from the data-holding institutions (cancer registries and health insurance providers) jointly defined the two seeds, which are never disclosed to any external party, including the TTP. This ensured a confidential, study-specific E-PIX^®^ configuration at the data holding institutions, enabling consistent application of Bloom filters, referred to as linkage keys, across the data-holding institutions.

### Results of the record linkage runs

The definitive record linkage was performed in the second quarter of 2025. The results of the PPRL of three cancer registries and claims data of three health insurance providers with the E-PIX^®^ are presented in Table 1.

A total of *N* = 1,437 stage IV NSCLC patients (target population) diagnosed during the recruitment period and insured by the participating health insurance providers were found following cohort matching within the participating cancer registries (see Fig. 1). Of these, *N* = 1,354 patients of the target population were successfully matched within the health insurance providers, resulting in a matching rate of 94.2%. The matching rates were similar across cancer registries, ranging from 93.6% to 97.8%. A total of *n* = 83 patients from the target population were not matched in the data set of the respective health insurance providers. The specific reasons for these non-matches cannot be determined conclusively; however, potential explanations are discussed in the following section.

**Table 1:** – Results of PPRL of cancer registry and claims data with E-PIX^®^ software solution of the population-based control group (pCG)

		Insurance provider 1	Insurance provider 2	Insurance provider 3	Overall
Cancer registry 1	Linkage key provided (N)	141	186	0	327
	Matches (N, %)	133 (94.3)	173 (93.0)	–	306 (93.6)
	Non-matches (N, %)	8 (5.7)	13 (7.0)	–	21 (6.4)
Cancer registry 2	Linkage key provided (N)	177	2	1	180
	Matches (N, %)	173 (97.7)	1 (50.0)	1 (100)	175 (97.8)
	Non-matches (N, %)	4 (2.3)	1 (50.0)	0 (0)	5 (2.2)
Cancer registry 3	Linkage key provided (N)	393	529	8	930
	Matches (N, %)	369 (93.9)	498 (94.1)	6 (75.0)	873 (93.9)
	Non-matches (N, %)	24 (6.1)	31 (5.9)	2 (25.0)	57 (6.1)

## Discussion

A comprehensive and valid evaluation of the *DigiNet* study can only be achieved through the utilization and linkage of cancer registry data and claims data. The feasibility of the proposed PPRL procedure as exact data linkage of cancer registry and claims data based on the KVNR without informed consent, was successfully demonstrated.

Historically, several record linkage approaches have been used in Germany to link cancer registry and claims data, each associated with specific methodological and practical challenges (March et al. 2018). Prior to the legal authorization for cancer registries to store the KVNR (introduced by the Cancer Early Detection and Registry Act in 2013), several German studies successfully applied probabilistic linkage based on encrypted personal identifiers. For instance, the linkage of data from the Cancer Registry of NRW, the AOK NordWest and the Disease Management Program for diabetes mellitus type 2 in order to investigate the cancer incidence (Kajüter et al. 2014). The implemented double encryption method is commonly used in German cancer registries to generate so-called control numbers and is based on the patient’s first name, last name, birth name, former name, date of birth, academic title, or other variables, depending on the cancer registry (Krieg et al. 2001). The quality of the linkage depends, e.g., on spelling mistakes or missing data, leading to incorrect or missing links between the data of a person and might influence the validity of the analysis and the interpretability of the results (Intemann et al. 2023). However, reported error rates in the literature range from 0.015% to 0.36% for homonym errors and from 0.18% to 1.81% for synonym errors in probabilistic linkage procedures based on control numbers (Giersiepen et al. 2010; Kajüter et al. 2014; Krieg et al. 2001). Similar approaches have also been applied in the German Mammography Screening Program (Fuhs et al. 2014; Giersiepen et al. 2010). These earlier studies proved that probabilistic linkage based on indirect identifiers for the record linkage of claims and cancer registry data in Germany is feasible but subject to limitations arising from data quality and completeness (Kajüter et al. 2014; Kollhorst et al. 2022).

More recently, the WiZen study linked nationwide data from the statutory health insurance provider AOK with three different state cancer registries to investigate the effectiveness of certification in oncology (Bobeth et al. 2023). As KVNRs were largely unavailable for the study period (2006–2017), WiZen relied on exact record linkage based on the postal code, birthdate, and gender as indirectly identifying variables, achieved a matching rate of 97.1%, as validated in a subgroup using the KNVR (Bobeth et al. 2023). Importantly, the authors emphasized that exact data linkage based on the KVNR should be considered as gold standard for future studies (Bobeth et al. 2023).

Against this background, the *DigiNet* study represents a next methodological step, implementing a KVNR-based exact PPRL approach across three German cancer registries and three statutory health insurance providers. This approach combines the precision of deterministic linkage with the privacy protection of irreversible encryption, thereby addressing methodological limitations of probabilistic linkage while maintaining a high level of data privacy protection.

Record linkage for research purposes presents a variety of challenges that can be categorized into the following dimensions: methodological, technical, structural, administrative, and data protection-related challenges. The significance of each of these varies depending on the study’s specific objectives and research questions. Notably, in the case of *DigiNet*, the methodological and technical challenges have already been addressed through the proposed PPRL procedure. As the primary focus is the record linkage of cancer registry and claims data in Germany, a brief overview of the landscape of cancer registries and health insurance providers will first be provided, before discussing the associated structural, administrative, and data protection-related challenges.

Population-based cancer registries are vital for the provision of reliable population level data on the national cancer burden in order to monitor and guide cancer surveillance and control (Coebergh et al. 2015; Piñeros et al. 2021). Further core objectives of population-based cancer registries are the evaluation of the quality of oncological care, e.g., adherence to current guidelines and quality indicators, and the advancement of health services research in oncology, e.g., by examining prognosis-related factors, such as tumor- and therapy-specific characteristics, and its impact on cancer recurrence and patient survival (Katalinic et al. 2023; Tucker et al. 2019). Therefore, all physicians in Germany who diagnose or treat tumor diseases are legally obliged to report such cases to the cancer registry of the state they are practicing in. Explicit informed consent from the patient is not required for cancer registration in Germany. However, patients may object to the collection of the clinical and/or the storage of their PII. The data format is standardized on the national level and precisely defines the parameters to be recorded (oBDS) (Arndt et al. 2020). Cancer registries in Germany are organized at the federal state level, with the exception of the Clinical-Epidemiological Cancer Registry Brandenburg-Berlin, which covers these two states (Katalinic et al. 2023).

In the German healthcare system, health insurance is compulsory, with a distinction made between statutory and private health insurance. Approximately 74.6 million Germany’s residents, representing about 87% of the population, are covered by a total of 95 different statutory health insurance providers (data as of 2024 (*vdek-Basisdaten des Gesundheitswesens in Deutschland 2024*, 2025)). Private health insurance is available only to those who are not required by law to have statutory health insurance, such as self-employed persons and employees with incomes above a certain threshold. Approximately 10% of the German population are privately insured (*vdek-Basisdaten des Gesundheitswesens in Deutschland 2024*, 2025). The data of the health insurance providers are collected for billing purposes of medical services.

Due to limited study duration and funding, it is usually not possible to collaborate with all German cancer registries and health insurance providers. Instead, studies often focus on cancer registries of specific model regions or statutory insurers covering a large proportion of the population, such as the *BARMER*, *Techniker Krankenkasse*, or the *AOK* (*vdek-Basisdaten des Gesundheitswesens in Deutschland 2024*, 2025). Therefore, claims data from private health insurance providers have rarely been considered in health care research in Germany (Hoffmann and Icks 2012). Depending on the research question, this may affect the representativeness of findings based on claims data (Hoffmann and Icks 2012; Kriwy and Mielck 2006).

Moreover, for studies spanning several German states, separate data use applications must be submitted to each cancer registry involved. The development of data flows and data use applications need to be adapted to state-specific legal frameworks, resulting in a complex and time-consuming application process. Likewise, for the claims data, an extensive data use application had to be submitted to the Federal Office for Social Security and the state-level supervisory bodies according to § 75 of SGB X, explaining in detail why the study cannot be carried out on the basis of individual declarations of informed consent, and specifying in detail the purposes for which the data will be used and all technical processes planned. The approval of the applications is always a case-by-case decision and depends on the research question of the study, the respective data flows, and the institutions involved. In *DigiNet*, the process from the preparation of the applications until full approval was granted by all involved partners took 2.5 years.

Although the matching rate in this study was high at 94.2% using exact PPRL based on the KVNR, a total of *n* = 83 cases of the target population could not be matched within the data sets of one of the participating health insurance providers. The specific reasons for these non-matches cannot be conclusively determined. One possible explanation may be differences in the filtering criteria applied by the data-holding institutions. In particular, health insurance providers filtered the target population based on the residential location relative to the three model regions, whereas cancer registries filtered the cases based on the treatment location. As such, some non-matches may involve individuals who received treatment in institutions within the model regions but resided in a neighboring federal state. Furthermore, the cancer registries may have recorded the health insurance provider at the time of the initial cancer diagnosis, while the patient might have changed providers prior to progression to stage IV disease, or the costs may have been reimbursed by social welfare under § 264 SGB V. This could result in a lack of corresponding records within the study period at the respective health insurance provider.

Since the KVNR is used for reimbursement processes in both the cancer registries and the statutory health insurances, any systematic documentation errors of this key matching variable should be rather unlikely. This assumption is supported with the relatively small number of non-matches. Furthermore, although not the focus of the manuscript, the *DigiNet* record linkage of the AOK claims data provided by the WIdO with the cancer registries, which was also based on the KVNR, yielded matching rates between 97.6% and 98.2%.

### Strengths and limitations

The PPRL concept presented has certain limitations. First of all, the success of this concept strongly depends on the availability and accurate documentation of the KVNR within the data-holding institutions, which was not the case for the AOK data provided by WIdO. Consequently, an alternative, institute-specific approach had to be applied instead of the proposed PPRL concept. Since 2013, the KVNR is also captured in cancer registries in Germany, as cancer registry reports are invoiced via the health insurance providers (Kollhorst et al. 2022). The IT infrastructure of the statutory health insurance providers and cancer registries must support the installation of external software. Additional costs for implementing the E-PIX^®^ may arise, e.g., if data-holding institutions contract an external service provider. Furthermore, the involvement of an independent TTP is required to perform the data linkage based on the linkage keys and to subsequently replace the keys with pseudonyms. The PPRL concept requires permission from the respective regulatory authorities of the statutory health insurance providers and the cancer registries. If a declaration of consent is available, a simplified data linkage concept could be applied, since, for example, the data-holding institutions could disclose identifying information, making encryption with the E-PIX^®^ unnecessary. In summary, this study demonstrates the feasibility of linking cancer registry and claims data without patient consent in Germany on the basis of an exact PPRL. This was possible as the study covers a period in which the KVNR is reliably recorded in the cancer registries.

### Lessons learnt and practical implications

The key lesson for future PPRL studies is that the record linkage between cancer registry and health insurance claims data based on the statutory health insurance number (KVNR) is feasible and that the documentation of the KVNR within the cancer registries is largely accurate.

The PPRL concept using the E-PIX^®^ software solution was thoroughly reviewed by data protection experts, who raised no objections regarding its compliance with data protection regulations. Installation and configuration of E-PIX^®^ were performed efficiently and without any major issues by all participating cancer registries and health insurance funds, following the implementation guidelines provided in the Supplementary File 1 (a German version is available upon request from the corresponding author). Importantly, the installation and configuration process does not require specific IT expertise, making the approach broadly applicable in different institutional settings. However, due to the heterogeneous IT infrastructure of the health insurance funds, the installation of external software may require additional administrative and financial resources. Furthermore, an independent trusted third party is required to perform the linkage using the encrypted identifiers, to remove the linkage keys after merging, and provide the merged dataset to the evaluating institutions.

For future PPRL studies, we strongly recommend involving all relevant stakeholders early in the planning process and submitting the data use applications as soon as possible. Moreover, test runs of the PPRL processes are highly advisable to help verify the correct installation and configuration of the E-PIX^®^. The coordination of processes and the approval procedures accounted for the majority of the study duration, whereas the actual technical implementation of the linkage concept was completed within a few weeks.

## Conclusions

The exact PPRL procedure based on the KVNR, developed as part of the innovation funds study *DigiNet*, has proven feasible while maintaining a high level of data privacy protection. This linkage facilitates case-specific evaluations of cancer care, such as the performance of cost-effectiveness analyses or the assessment of the implementation of guideline-based therapies. The concept can be applied to other settings with real-world data sources working with uniquely identifying variables such as personal identity numbers. The PPRL procedure presented here is based on the publicly available E-PIX^®^ software solution, and can thus serve as blueprint for other studies requiring case-specific linkage of different data sources without compromising data privacy.

## Supplementary Information

Below is the link to the electronic supplementary material.

Supplementary Material 1

## Data Availability

Not applicable.
