# Supplementary material for: Concept and feasibility of privacy-preserving record linkage of cancer registry data and claims data in Germany: results from the DigiNet study on stage IV non-small cell lung cancer
Source: J Cancer Res Clin Oncol. 2025 Dec 4;152(1):6. doi: 10.1007/s00432-025-06384-7 (PMC12678689; doi:10.1007/s00432-025-06384-7)
Supplement: Supplementary file 1 — Supplementary Material 1 [file 432_2025_6384_MOESM1_ESM.pdf]

Guideline for installation and configuration of E-PIX®

E-PIX®

Enterprise Identifier Cross-Referencing

Created by:

Lizon Fiedler-Lacombe,

Pia Naumann

Reviewed by:

Dr. Anika Kästner,

Dr. Martin Bialke,

Christopher Hampf

DATE: 25.06.2025

VERSION 2.1

Table of content

1. Introduction 3

2. Installation of E-PIX® with “Docker for Windows” 4

3. Configuration of E-PIX® via User Interface 5

3.1 Creating the domains and sources 5

3.2 Creating a data source (Safe data source) 6

3.3 Creating a domain (Matching-Domain) 7

3.3.1 Creating a domain manually 7

3.3.1.1 Creating a domain: Tab Settings 8

3.3.1.2 Creating a domain: Tab Person fields 9

3.3.1.3 Creating a domain: Tab Preprocessing 10

3.3.1.4 Creating a domain: Tab Matching 10

3.3.1.5 Creating a domain: Tab Privacy 12

3.3.2 Creating a domain via XML file 14

4. Encrypting the PII to Bloom filters 19

5. Exporting the PII and Bloom filters via export function 21

1. Introduction

This guideline for the installation and configuration of the E-PIX® was developed as part of the DigiNet project and illustrates one of several use cases for the E-PIX® services. Further details on the DigiNet project can be found here:

https://www.ths-greifswald.de/projekte/diginet/

In the specific use case of the DigiNet project, data from two sources (cancer registries and health insurance providers) are merged using health insurance numbers (KVNR), without these numbers ever leaving the respective institutions or being disclosed to any third parties. For this purpose, Bloom filters are used to enable Privacy-Preserving Record Linkage (PPRL).

The first section of this guideline addresses the installation of the E-PIX® on the operating system Windows. Subsequently, the configuration of the E-PIX® domain and the encrypting of person identifying information (PII), such as the health insurance number (KVNR) to Bloom filters, is described. The configuration steps presented serve as example and must be adapted to the specific context of each project.

A detailed description of all services of the E-PIX® can be found in the E-PIX® manual:

http://www.ths-greifswald.de/e-pix/handbuch

In the E-PIX® manual, background, definitions, concept and functionalities of all services are described in detail in the Chapters 1-2.

The manual chapter references within this guideline are provided for those interested in more in-depth information about the software solution. Reading the referenced manual chapters is not required. This guideline alone is sufficient for the installation and configuration of the E-PIX® to perform PPRL based on the KVNR.

If you have any further questions regarding the installation and configuration of the E-PIX®, please contact the Independent Trusted Third Party (TTP) in Greifswald at kontakt-ths@uni-greifswald.de or use the contact form of the TTP available at https://www.ths-greifswald.de/kontakt.

2. Installation of E-PIX® with “Docker for Windows”

Please note that a user with admin rights is required to install and start the software solution. E-PIX® is operated as a Docker-Container by default. For the installation, you must first install the current version of the Docker and Docker-Compose[1] on the target system. To do so, you can find the instructions under the following links:

https://docs.docker.com/install

https://docs.docker.com/compose/install/

Further information on the system requirements can be found in Chapter 2.2.1 of the manual.

Once you have downloaded and installed the Docker Desktop app successfully, start the app on your target system. Then, you can download the E-PIX® via the THS website using the following link:

https://www.ths-greifswald.de/forscher/e-pix/#download

Unpack the archive at the desired location. After that, the E-PIX® can be started via Docker-Compose. The steps required for this on a Windows-operated system are described below:

Start the Windows Console CMD with admin rights and change to the selected directory (contains the docker-compose.yml file). To ensure that E-PIX® can be started on Windows without any problems, set the parameter #WF_MARKERFILES = AUTO to FALSE in the file envs/ttp_commons.env and remove the hash (#) in front of it. Now, the configuration parameter should look as follows:

„WF_MARKERFILES = FALSE“

By using the following command, you can start the E-PIX® via Docker-Compose in the console:

„docker-compose up“

It may take a few minutes to start the software solution. E-PIX® has been installed successfully when the following command is displayed:

„Wildfly 26.1.2. Final […] started in …“

Finally, you can use the E-PIX® on your target system. To do this, open a new tab in your web browser and enter the following URL:

„localhost:8080/epix-web“

Further information on downloading and starting the service via Docker can be found in Chapter 2.2 of the manual.

[1] Docker-Compose is now integrated into the Docker Desktop app, so only a single app needs to be installed on the computer.

3. Configuration of E-PIX® via User Interface

Testing E-PIX®

To explore E-PIX®, you may use the graphical user interface on the TTP demo system, accessible via the following link:

https://demo.ths-greifswald.de/epix-web/

Please do not enter any real data, as all entries are visible to other website visitors. The system is reset daily, and all previously entered data will be deleted.

By default, the E-PIX® you have installed can be accessed at <URL>":8080/epix-web/". If E-PIX® is running on your local device, the service can be accessed at http://localhost:8080/epix-web/, for example.

E-PIX® manages the entered data in domains. Therefore, a domain must be created to enter the PII, e.g. the health insurance numbers (KVNR). This enables the PII to be encrypted to Bloom filters. A safe data source and an identifier-domain must be specified for each domain (see manual Chapter 3.1 and 3.2). The E-PIX® has a default identifier-domain that can be used for this purpose (MPI) (see Figure 1).

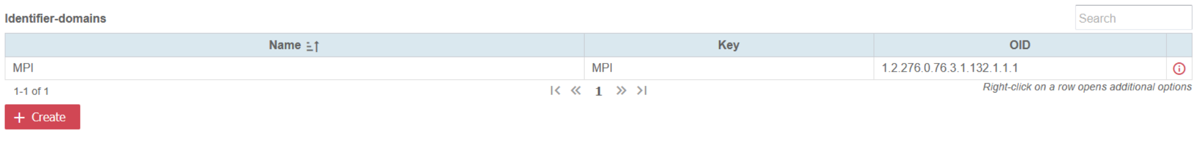

Figure 1 – Identifier-Domain

3.1 Creating the domains and sources

In the left menu, click under **"Settings"** on **"Domains/Sources/Identifiers"** (see Figure 2).

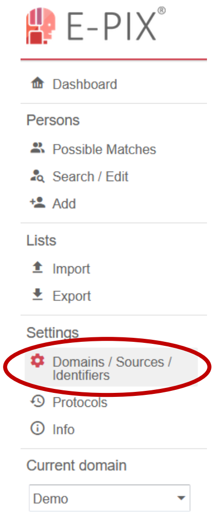

Figure 2 – E-PIX® menu

3.2 Creating a data source (Safe data source)

Then, click under **"Data sources"** on **"Create"** (see Figure 3).

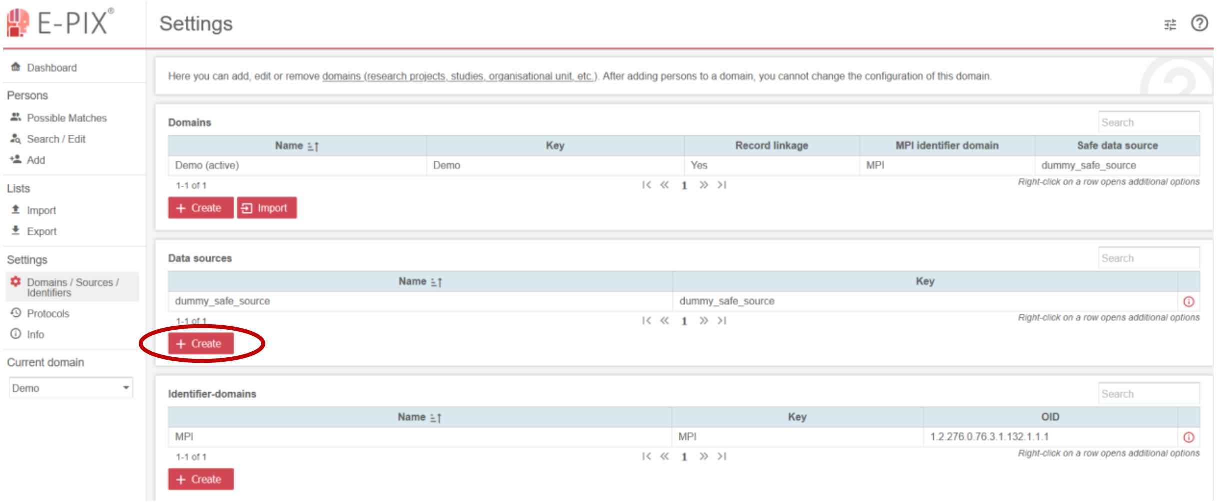

Figure 3 – Interface "Settings" for Creating a safe data source

Now, fill in the tab **"New data source"** as follows:

- **Name**: example "cancer registry" or "insurance provider" (freely selectable)
- **Key**: example "cancer registry" or "insurance provider" (freely selectable)
- **Description**: freely selectable (user-defined text; leave blank if necessary)

(see manual Chapter 4.1 (Explanation of the term "source" in Chapter 3.1))

Then, click on **"Create"** (see Figure 4).

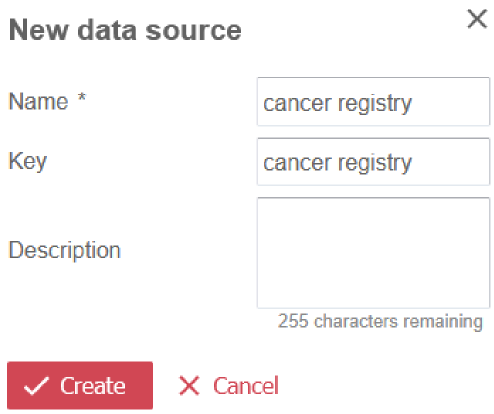

Figure 4 – Tab creating a "New data source"

Once a **"Safe data source"** has been created successfully, the domain can be created (see manual Chapter 4.3).

3.3 Creating a domain (Matching-Domain)

There are two options for creating a domain:

1) The domain is created manually according to the configuration on **"Create"** (see Chapter 3.3.1 **Creating a domain manually**).

2) The domain is created using a preconfigured XML file by clicking on **"Import"** (see Chapter 3.3.2 **Creating a domain via XML file**).

Both practices are explained in detail in the sections 3.3.1 and 3.3.2 below.

3.3.1 Creating a domain manually

Please ensure that all required tabs for the domain are completed before clicking the **"Add"** button to create the domain.

Click under **"Domains"** on **"Create"** (see Figure 5).

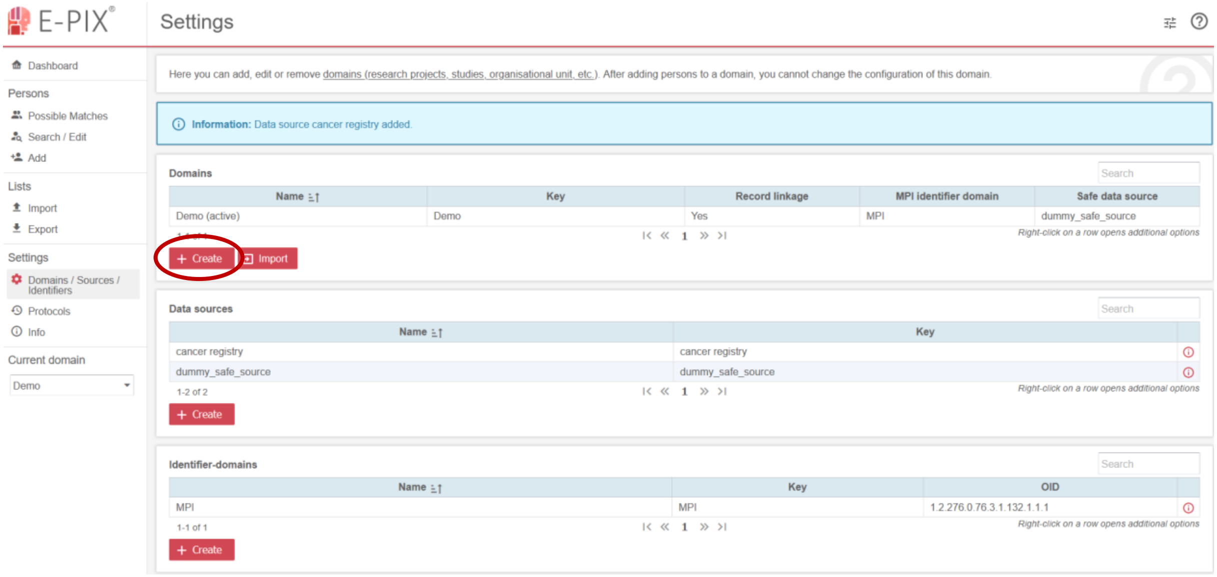

Figure 5 – Interface "Settings" for creating a domain

3.3.1.1 Creating a domain: Tab Settings

Fill the tab **"Settings"** as follows (see Figure 6):

- Name: example "population-based comparison group"
- Key: example "population-based comparison group"
  (is generated automatically if the field is left blank)
- Description: example "Generating a Bloom filter for the health insurance number of the patients in the population-based comparison group" (user-defined text; leave blank if necessary)
- Safe data source: "cancer registry" or "health insurance provider" (previously created data source)
- **Send notifications from the web interface:** Do not set a check mark

Master Patient Index (MPI)

- **Generator:** EAN13Generator
- **Prefix:** 1001
- **Identifier domain:** MPI

Performance

- **Activate parallel matching after:** 1,000
- **Threads for parallel matching:** 4
- **Limit search to matching fields (reduces memory usage but prevents search for other fields):** Do not set a check mark

Further information is provided in the manual in Chapter 4.3.1.

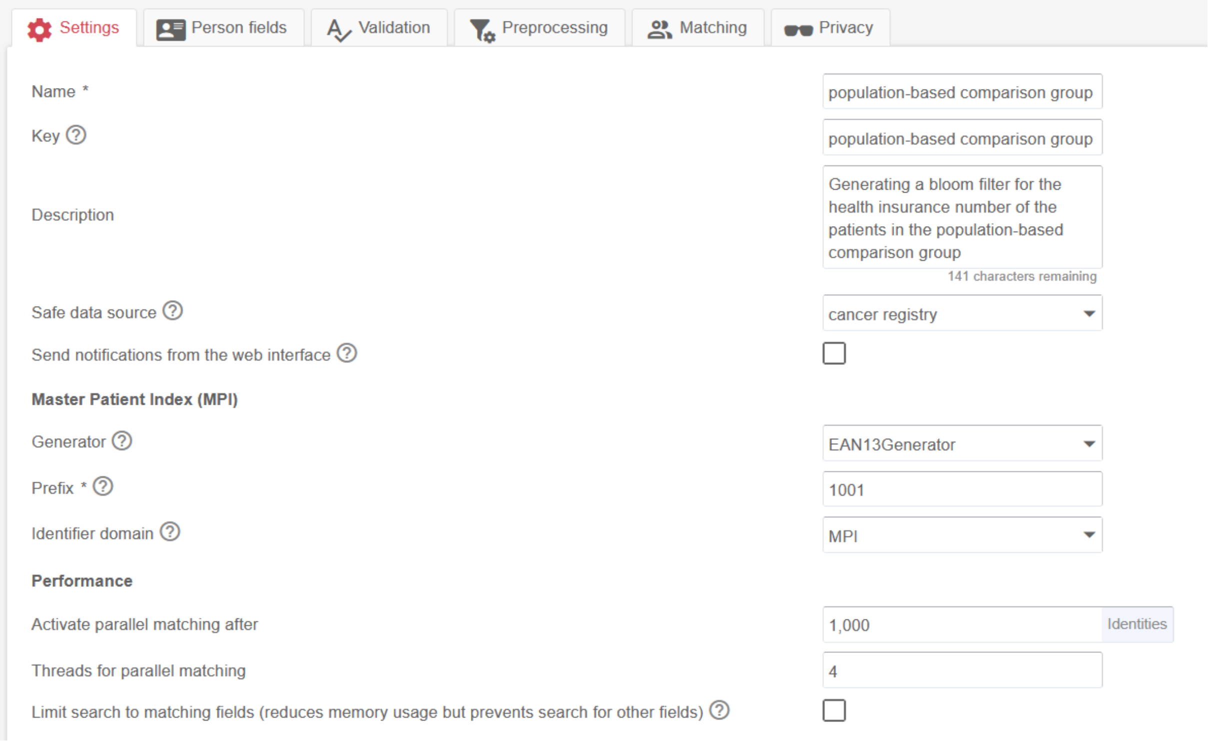

Figure 6 – Tab "Settings" during Creating a domain

3.3.1.2 Creating a domain: Tab Person fields

Fill in the tab **"Person fields"** as follows:

1) Click on the selection menu **"Mandatory fields"** and select **"Field 1"** by ticking the box (see Figure 7).
2) Remove all other check marks that are set by default.
3) Close the window again by clicking outside the window area.

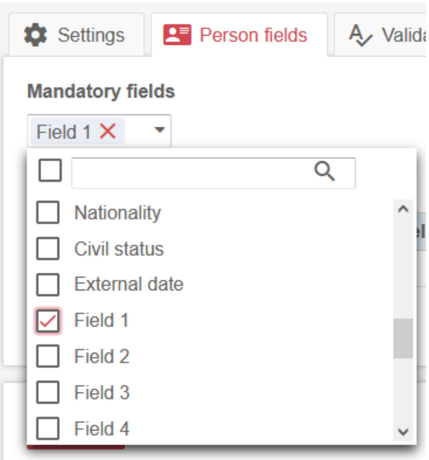

Figure 7 – Tab "Person fields" for selecting the mandatory fields

4) Now, click under **"Additional fields"** on **"Add additional field"**.
5) Please fill in each **"Field"** and **"Label"** (see Figure 8) und then click on **"Add"**.

Repeat this for all additional fields. Therefore, enter the following values:

Field	Label
Field 1 (50 chars)	KVNR
Field 8 (1000 chars)	Bloom filter

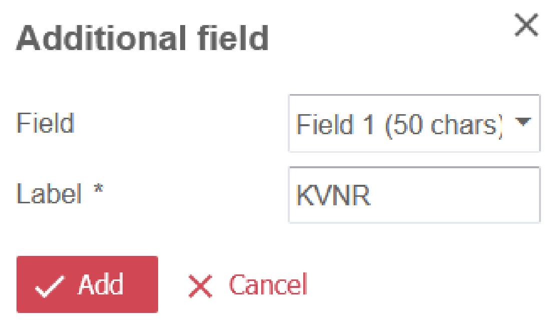

Figure 8 – Tab "Person fields" for creating the additional fields

Now, the tab **“Person fields”** should look as follows:

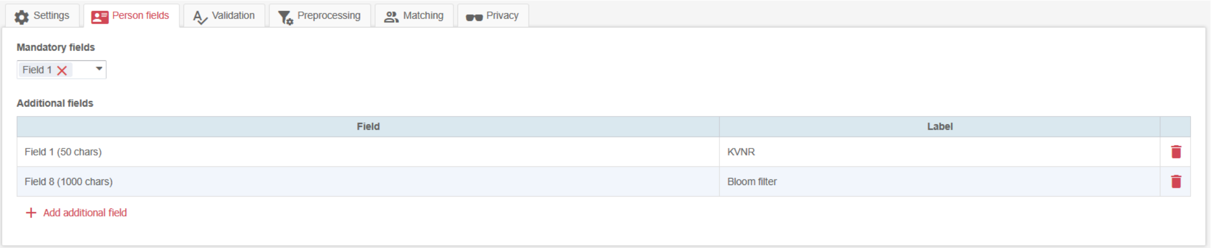

Figure 9 – Tab "Person fields" for creating a domain

Further information is provided in the manual in Chapter 4.3.2.

3.3.1.3 Creating a domain: Tab Preprocessing

Fill in the tab **“Preprocessing”** as follows (see Figure 10):

Remove all fields from the table **“Fields with preprocessing”** by clicking on the garbage can icon.

The tab **“Preprocessing”** should look as follows:

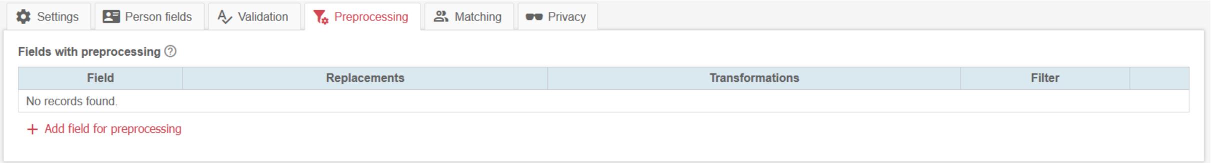

Figure 10 – Tab "Preprocessing" for creating a domain

Further information is provided in the manual in Chapter 4.3.4.

3.3.1.4 Creating a domain: Tab Matching

Fill in the tab **“Matching”** as follows (see Figure 12):

- **Perform record linkage:** Yes
- **Check if given identifying data matches equally well with other persons besides the identifier`s person:** Do <u>not</u> set a check mark
- **Threshold for possible Match:** 2.99
- **Threshold for automatic Match:** Set check mark at “never”

Remove all existing **“Matching fields”** and **“Reasons for resolving possible match”** by clicking on the garbage can icon.

Now, click on **“Add matching field”**.

Fill in the **“Matching field”** as follows (see Figure 11):

- **Field:** Field 1
- **Algorithm:** DeterministicAlgorithm
- **Mode:** Text
- **Blocking threshold:** 0%
- **Matching threshold:** 100%
- **Weighting:** 1.0

After that, click on **“Save”**.

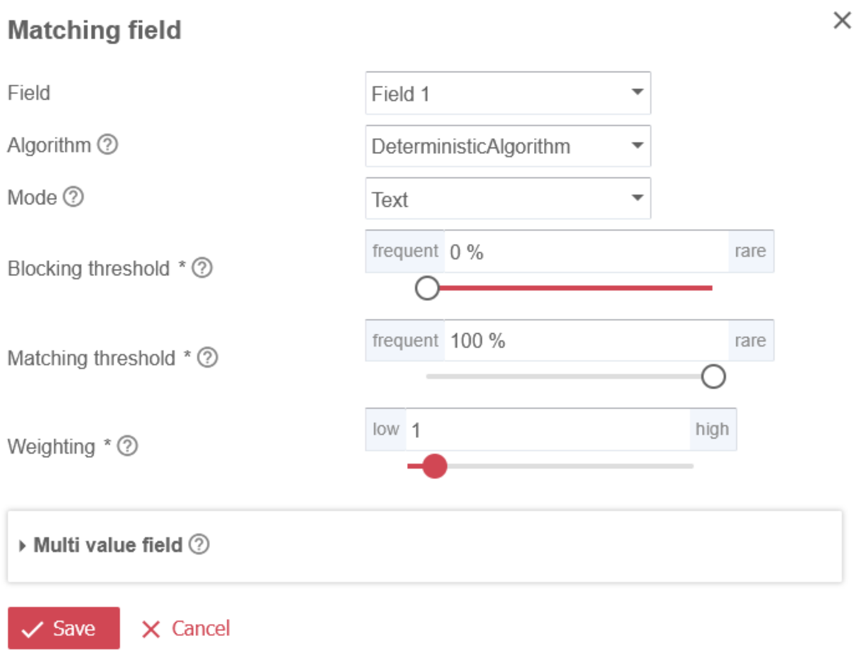

Figure 11 – Tab "Matching" to add a new matching field

The tab **“Matching”** should look as follows:

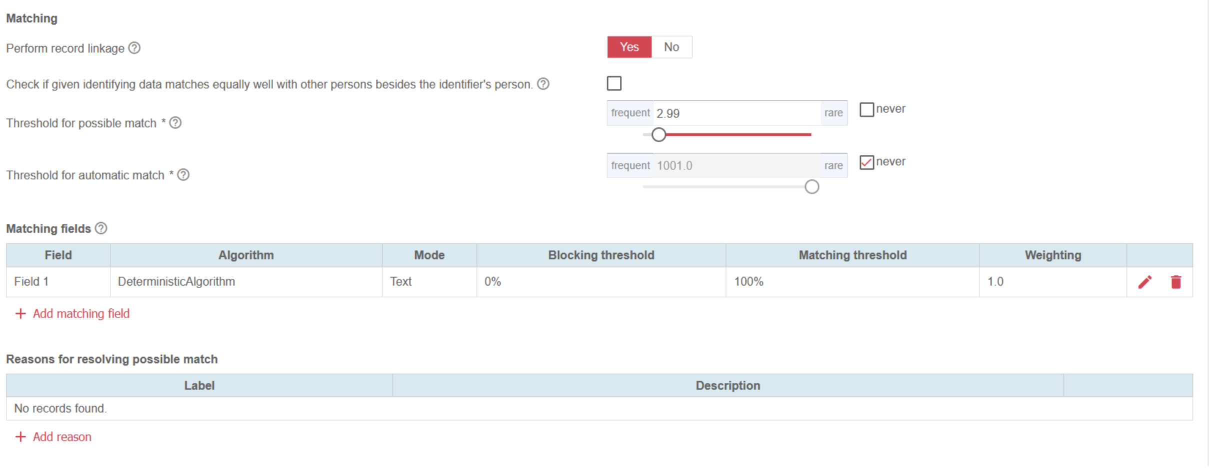

Figure 12 – Tab "Matching" for creating a domain

Further information is provided in the manual in Chapter 4.3.5.

3.3.1.5 Creating a domain: Tab Privacy

Fill in the tab **"Privacy"** as follows:

Click under **"Bloom filter"** on **"Add bloom filter".**

Fill in the fields of the **"Bloom filter"** as follows (see Figure 13)[2]:

- **Algorithm:** RandomHashingStrategy
- **Alphabet:** ABCDEFGHIJKLMNOPQRSTUVWXYZ0123456789
- **Length:** 300
- **Length of n-grams:** 2
- **Bits per n-gram:** 20
- **Amount of xor-folds:** 0
- **Balanced bloom filter:** Set a check mark
- **Seed:** x,xxx,xxx,xxx
- **Storing field:** Field 8

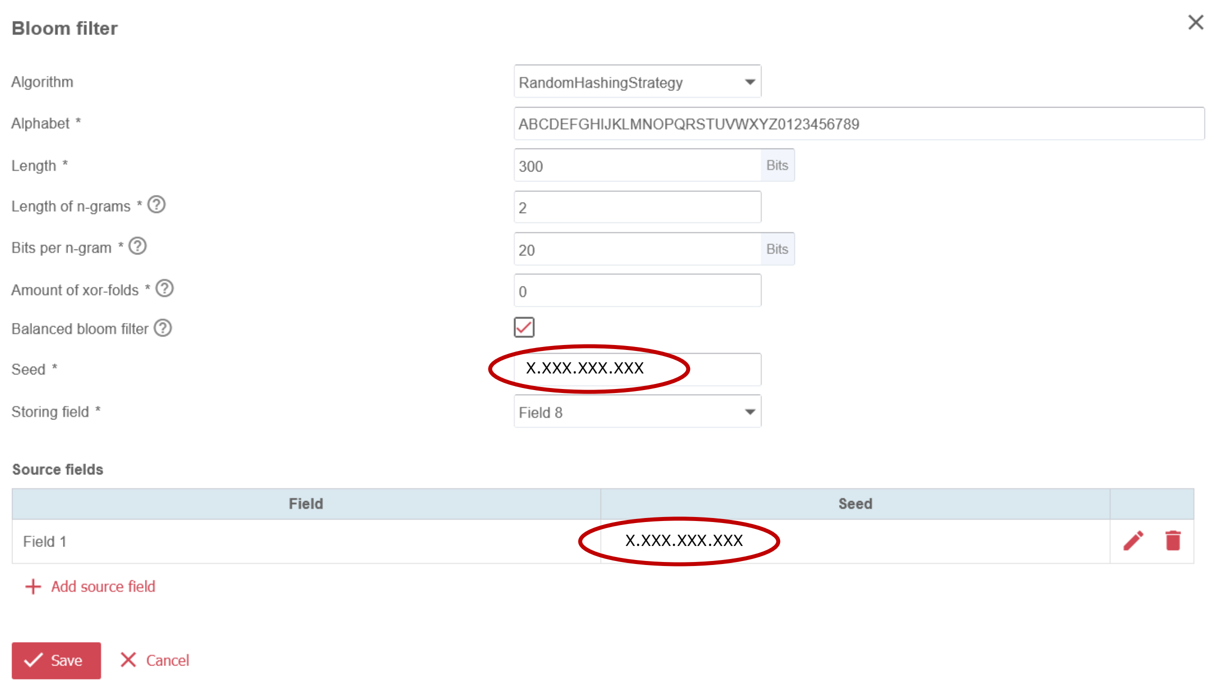

Figure 13 – Tab "Privacy" to add a Bloom filter

[2] The seed is a unique, selectable numerical code between 8 and 18 digits. Reproducibility of Bloom filters requires the use of the same seed.

Click under **“Source fields”** on **“Add source field”**.

Fill in the **“Source field”** as follows (see Figure 14)[3]:

- **Field:** Field 1
- **Seed:** x,xxx,xxx,xxx

Then, click on **“Save”.**

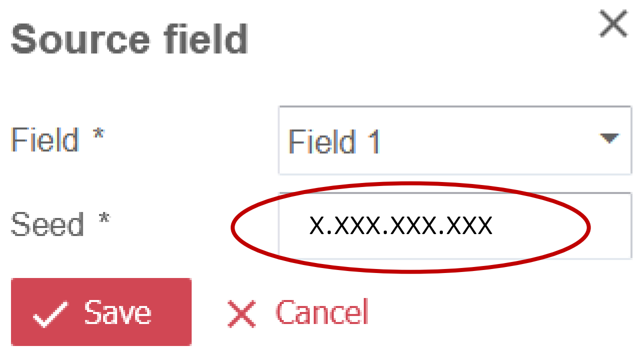

Figure 14 – Tab "Privacy" to create a source field

The **“Bloom filter”** should now look as shown in Figure 13.

After you have added the source field and filled in all fields correctly, click on **“Save”**.

The tab **“Privacy”** should now look as shown in Figure 15.

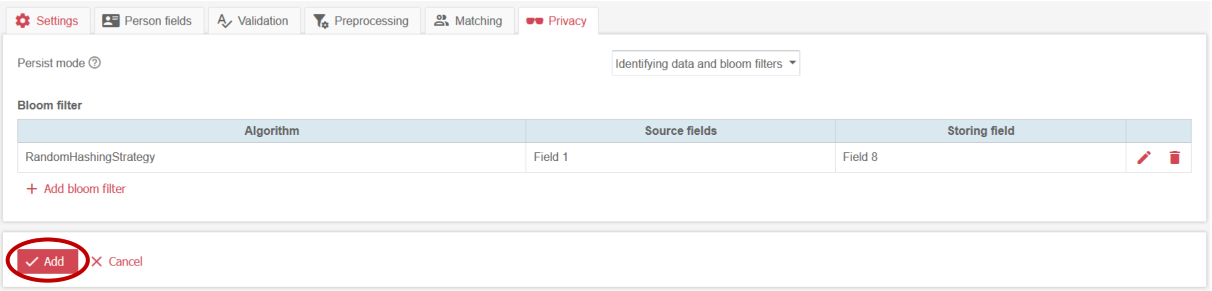

Figure 15 – Tab "Privacy" for creating a domain

Further information is provided in the manual in Chapter 4.3.6.

Configuration of the domain is now complete. Finally, click on **“Add”** to save the configuration of the domain (see Figure 15).

---

[3] A second seed between 8 und 18 digits is required for the source field, which must be different from the seed of the Bloom filter.

The E-PIX® settings interface should now look as follows:

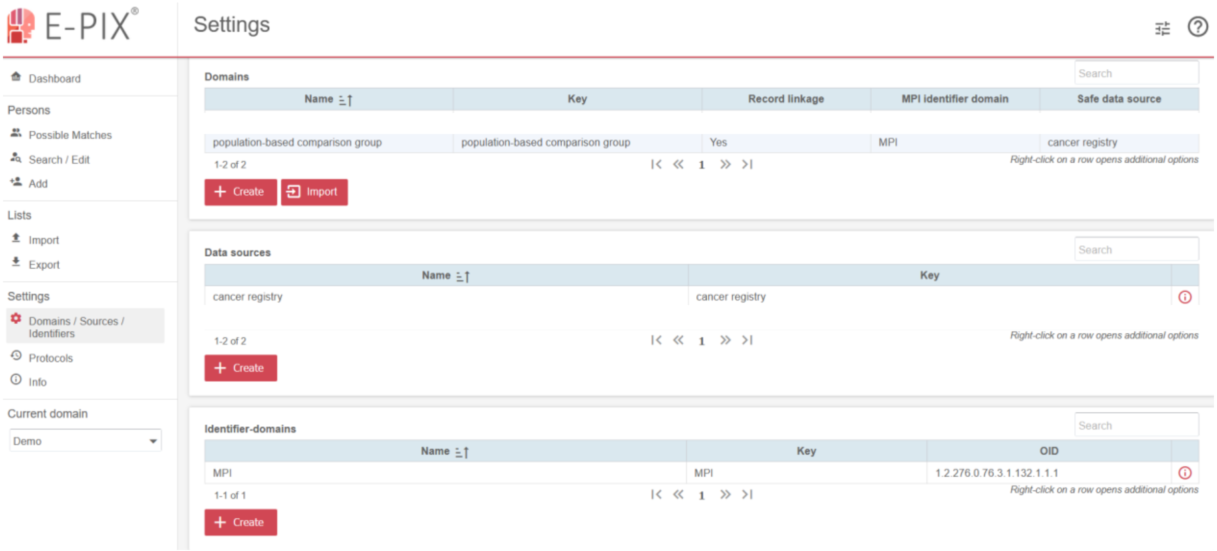

Figure 16 – Interface "Settings" of the E-PIX® after configuration of the domain

3.3.2 Creating a domain via XML file

Since January 2024, the automated configuration option is available using an XML file. **To use this alternative, please follow all instructions including Chapter 3.2 Creating a data source (Safe data source).**

Once you have created the **"Safe data source"**, you can import the XML file to configure the domain. Therefore, please click under **"Domains"** on **"Import"** (see Figure 17).

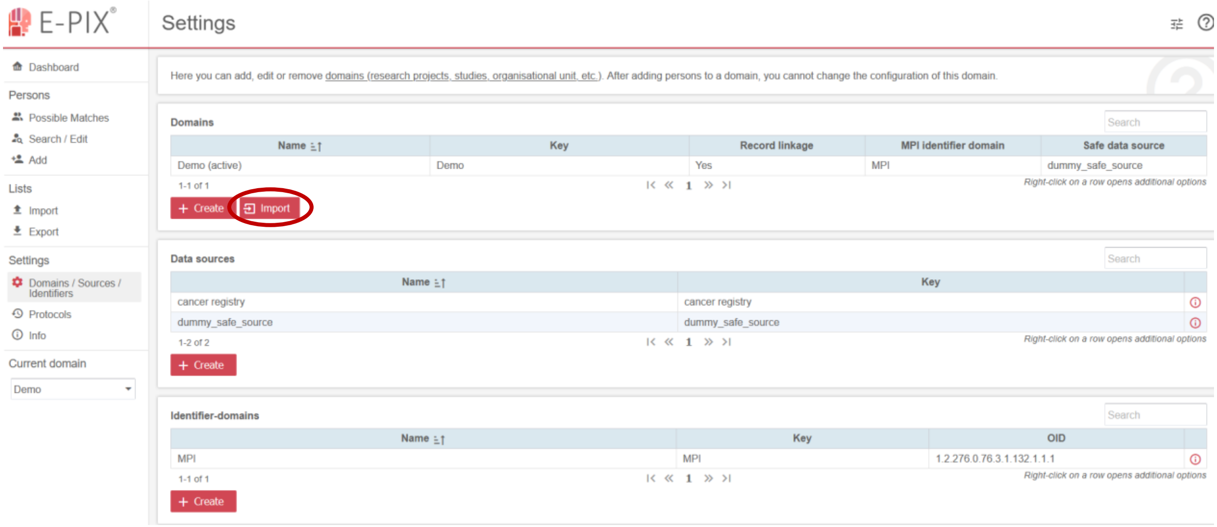

Figure 17 – Interface "Settings" to import a domain

Search your file system for the XML file that you received from the Independent Trusted Third Party by e-mail.

Click on the XML file and then on **"Open"** to import the XML file into the domain.

After successful import of the XML file, the domain configuration opens automatically with the pre-filled tabs and fields.

The following minimal adjustments must be completed manually:

Under the tab **“Settings”**, the **“Name”** of the domain must be added (see Figure 18).

Check that the details under **“Safe data source”** and **“Identifier domain”** have been selected correctly and adjust them if necessary (see Figure 18).

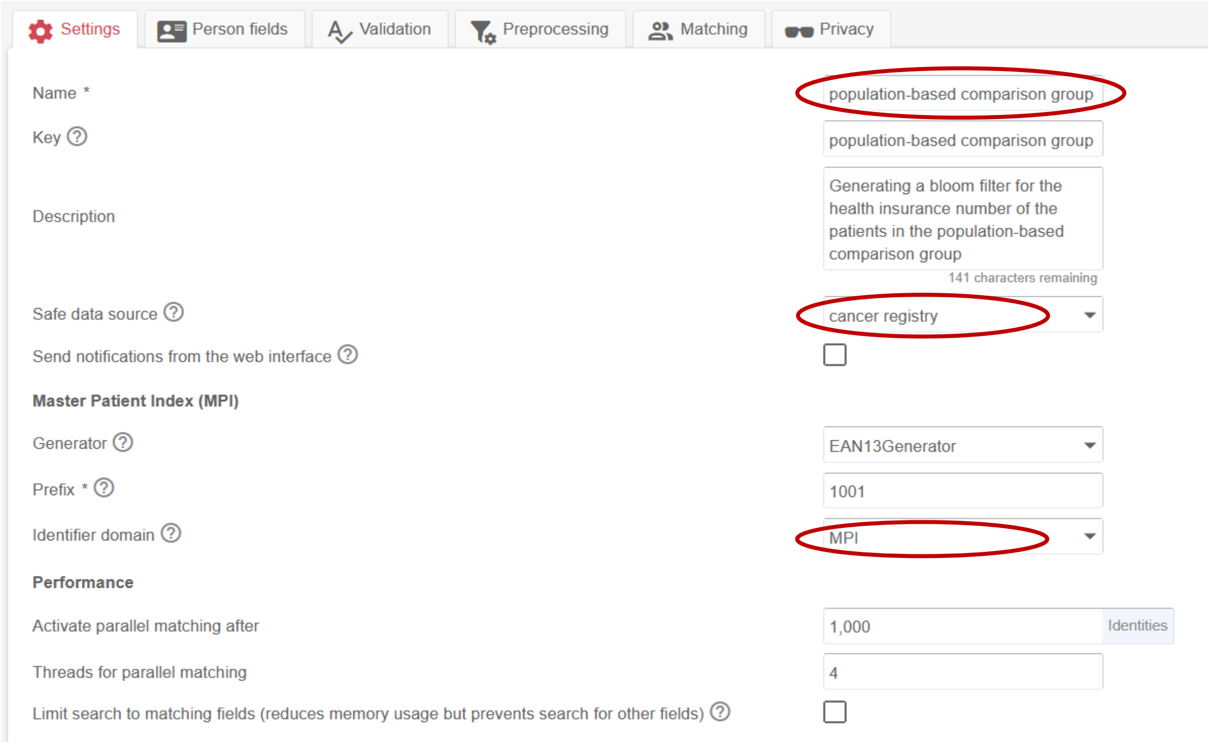

Figure 18 – Tab “Settings” during creating a domain

The tabs **“Person fields”**, **“Preprocessing”** and **“Matching”** are already filled out completely. You do not need to make any adjustments within these three tabs.

To add the two seeds for the Bloom filter and the source field, please navigate to the tab **“Privacy”**. Click on the pencil icon to edit the **“Bloom filter”** (see Figure 19).

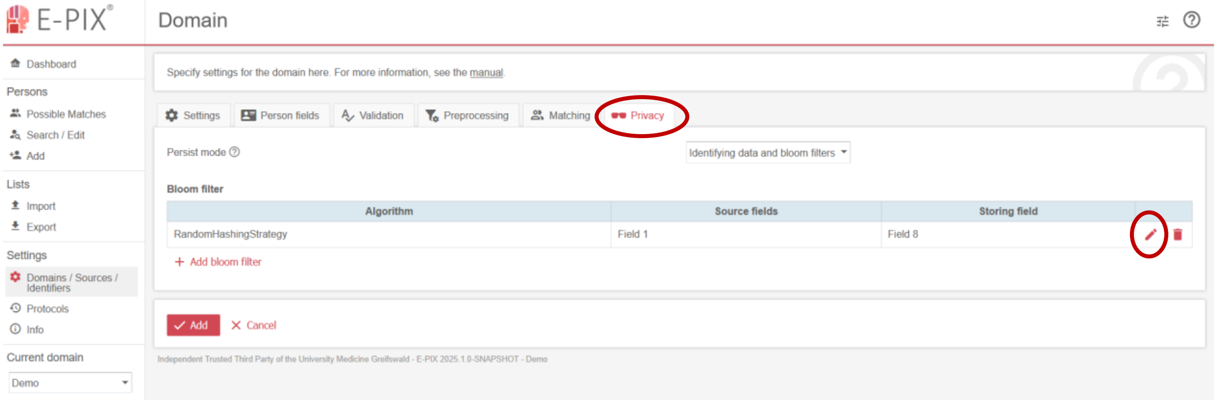

Figure 19 – Tab "Privacy" during creating a domain

A window opens in which you can enter the seed for the **"Bloom filter"** (see Figure 20)[4].

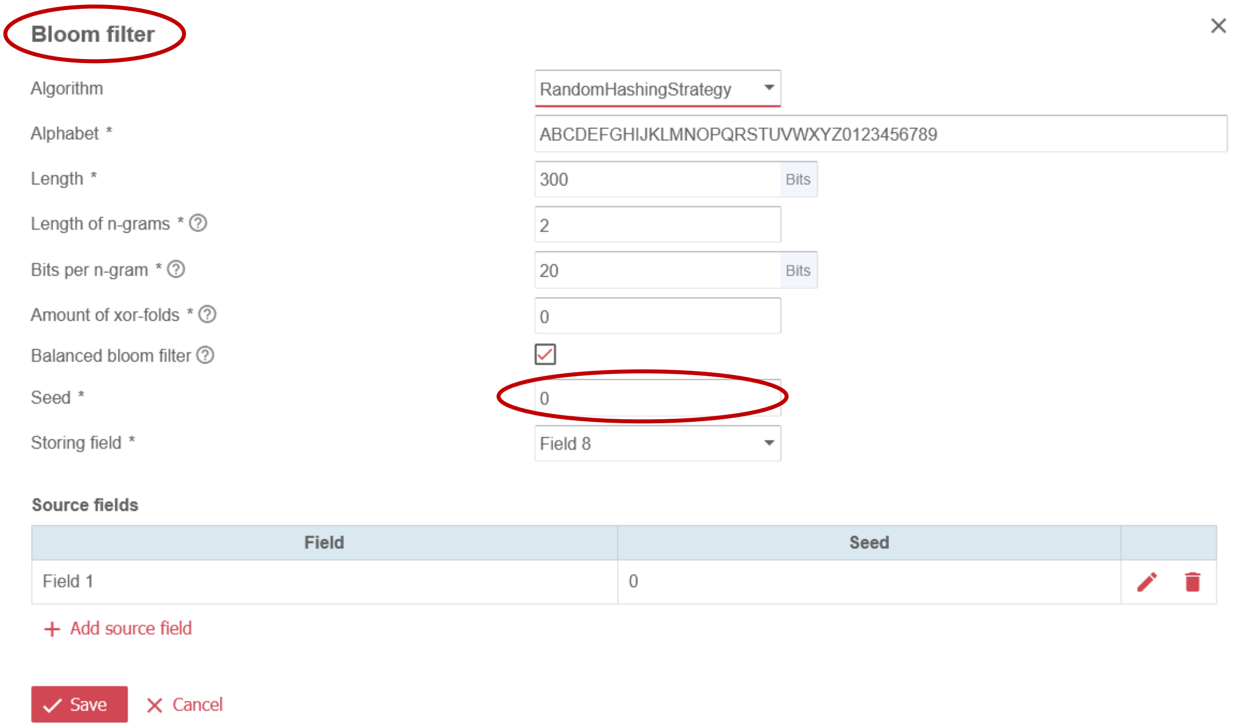

Figure 20 – Tab "Privacy" to add a Bloom filter

Note: When importing the XML file, the seed of the Bloom filter is set to "0" automatically, so you must add the required value at this point.

[4] The seed is a unique, selectable numerical code between 8 and 18 digits. Reproducibility of Bloom filters requires the use of the same seed.

To add the seed for the **“Source field”**, please click under **“Source fields”** on the pencil icon (see Figure 21).

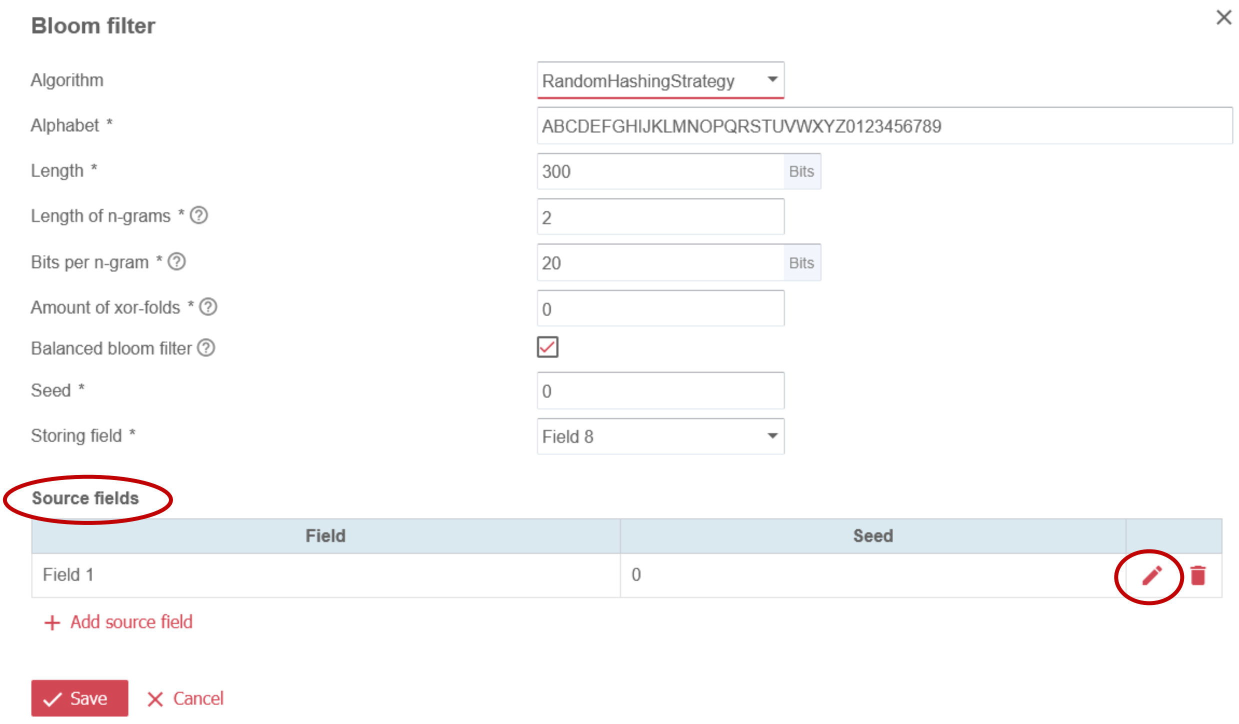

Figure 21 – Tab "Privacy" to create a source field

A window opens in which you can enter the seed for the **“Source field”** and then click on **“Save”** (see Figure 22)[5].

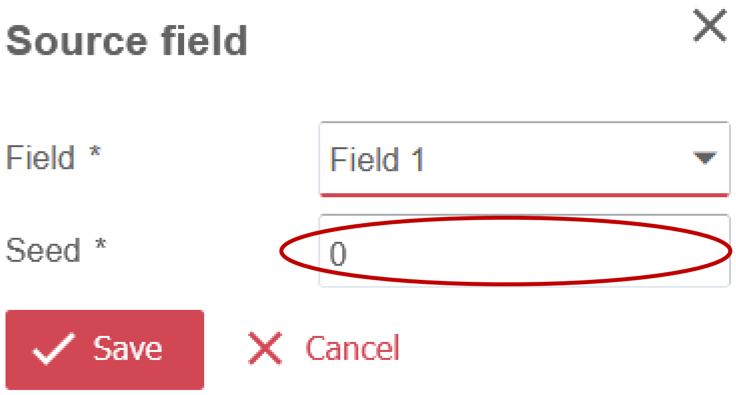

Figure 22 – Tab "Privacy" to create a Source field

Note: When importing the XML file, the seed of the bloom filter is set to “0” automatically, so you must add the required value at this point.

[5] A second seed between 8 and 18 digits is required for the source field, which must be different from the seed of the Bloom filter.

Once you have added the two seeds, please click under **“Bloom filter”** on **“Save”** (see Figure 23).

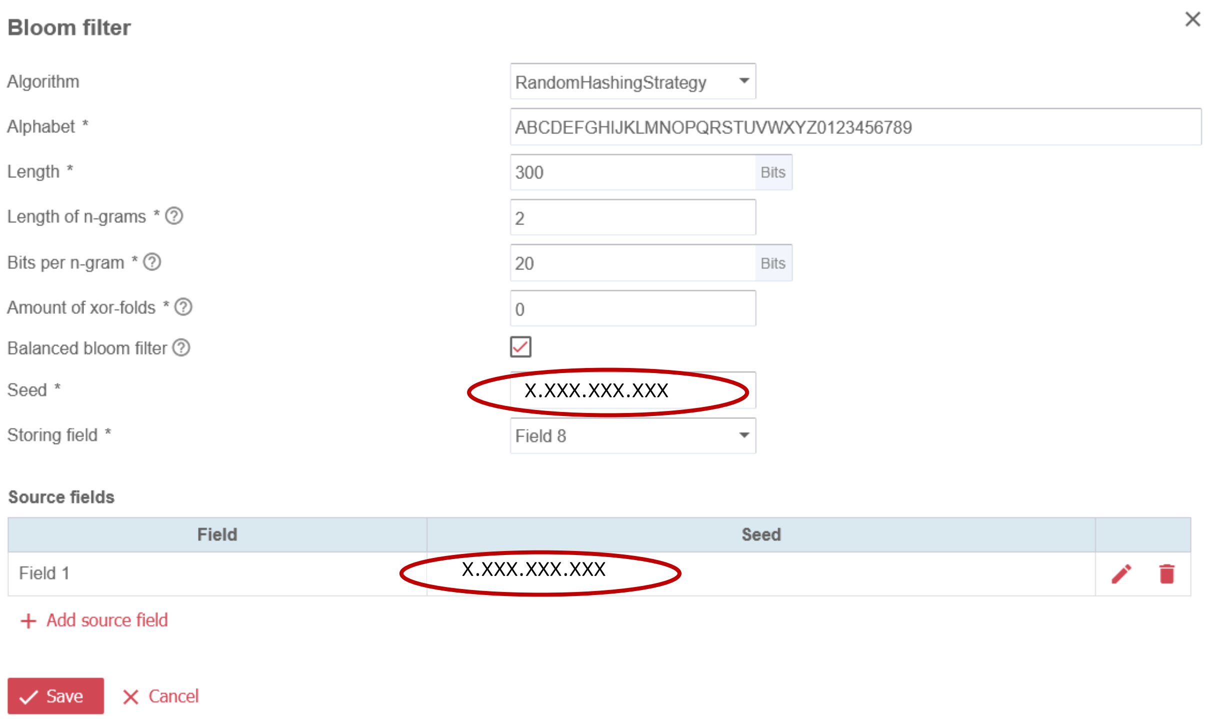

Figure 23 – Tab "Privacy" ta add a bloom filter

Finally, click on **“Add”** (see Figure 24). The domain settings have been successfully completed.

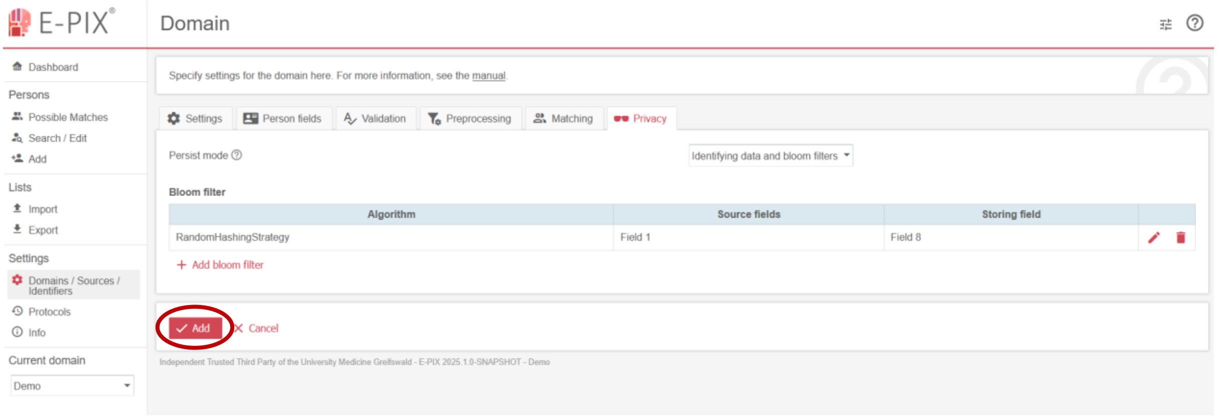

Figure 24 – Tab "Privacy" for creating a domain

4. Encrypting the PII to Bloom filters

Note: Please ensure that no PII is imported before the configuration is fully completed. Otherwise, the E-PIX® must be restarted and reconfigured.

After creating the safe data source and the (matching) domain, you can encrypt the corresponding PII, such as the health insurance number (KVNR), into Bloom filters. Therefore, a CSV file with the PII is imported into the E-PIX®. In the following, it is assumed that the CSV file only contains one column with the corresponding PII. After that, the E-PIX® generates a CSV file with the PII and the generated Bloom filters.

To import the CSV file, please proceed as follows:

1) In the left menu, click under **"Lists"** on **"Import"** (see Figure 25).

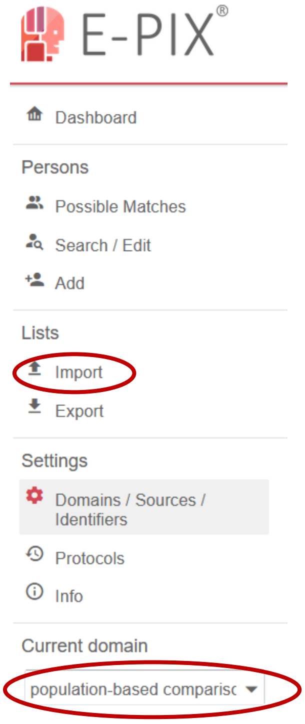

Figure 25 – E-PIX® menu

Note: If you are working in the demo system or have already created several domains, please make sure that the domain you need is selected under **"Current domain"** (see Figure 25).

2) Set a check mark on **"The list contains a header row with column names"**, if the CSV file you would like to upload has a header row or column description (see Figure 26).

3) Click on **“Choose file”** and upload the CSV file.

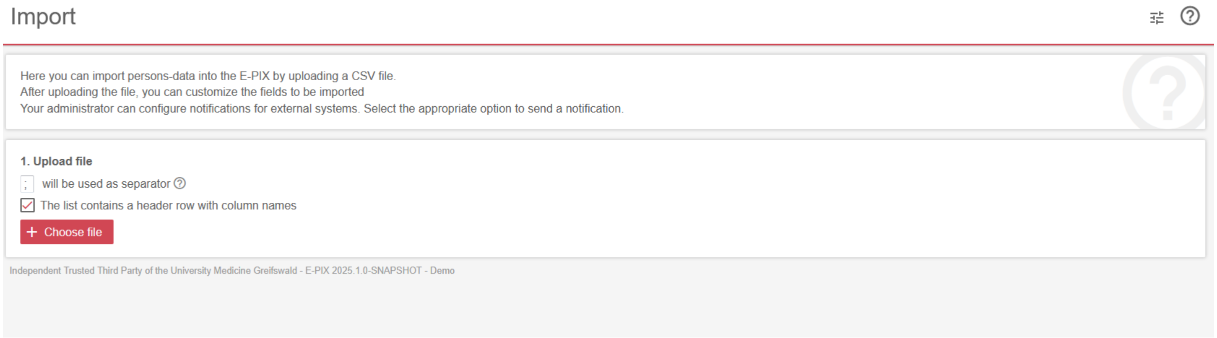

Figure 26 – Import via the interface

Under **“Select fields and set type”** you can see the first data set of the file (see Figure 27)[6].

4) Check all fields that you would like to import.

5) Select a data source. This corresponds to your **“Safe data source”**.

6) Finally, click on **“Import”**.

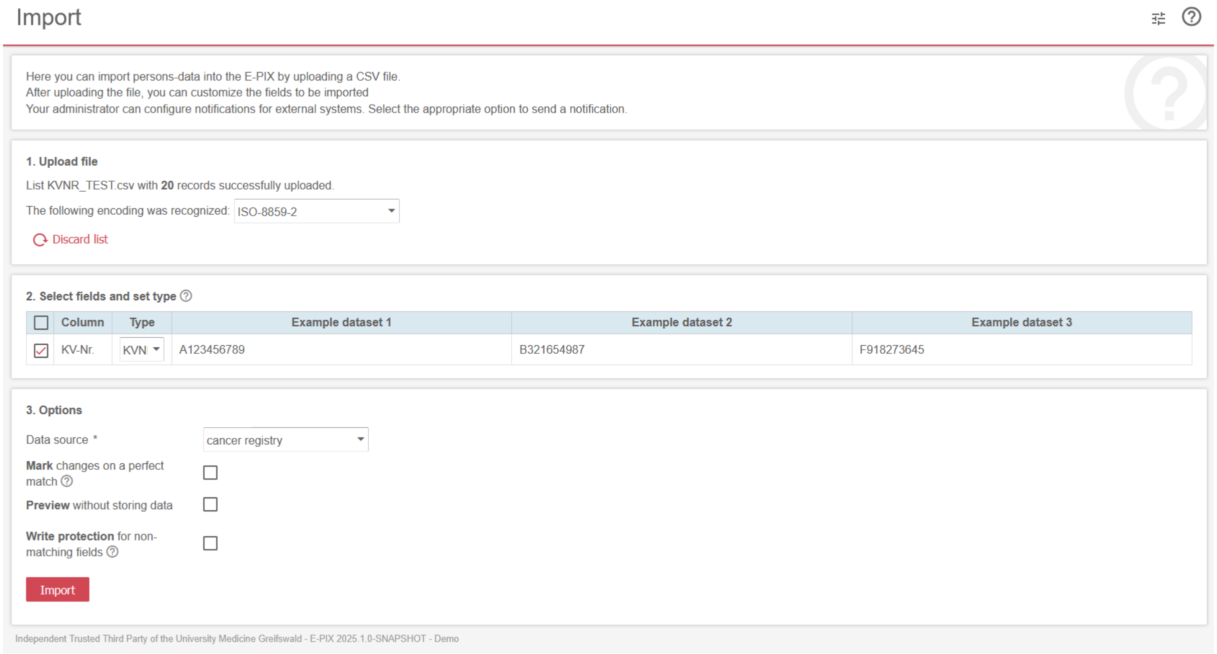

Figure 27 – Importing data into the E-PIX®

After a successful import (see Chapter 4), you can download the result by clicking on **“Download CSV”**. You will receive a CSV file with the PII and the generated Bloom filters as well as other variables (match status, MPI, data source, created by, last edited at) (see Figure 28)[6].

Further information is provided in the manual in Chapter 8.7.

[6] The health insurance numbers shown here are fictitious and do not correspond to real individuals.

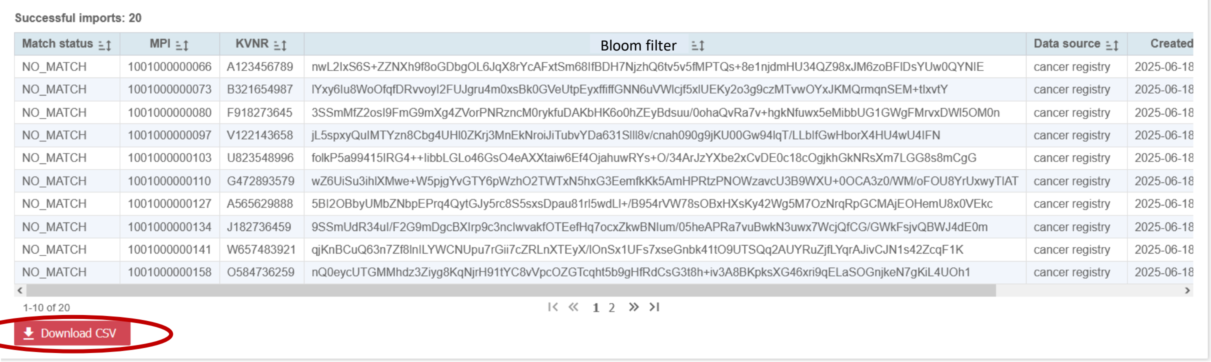

Successful imports: 20

Match status	MPI	KVNR	Bloom filter	Data source	Created
NO_MATCH	1001000000066	A123456789	nwL2IxS6S+ZZNXh9f8oGDbgOL6JqX8rYCAFxtSm68IfBDH7NjzhQ6tv5v5fMPTQs+8e1njdmHU34QZ98xJM6zoBFIDsYUw0QYNIE	cancer registry	2025-06-18
NO_MATCH	1001000000073	B321654987	lYxy6lu8WoOfqfDRvvoyl2FUJgru4m0xsBk0GVeUtpEyxffiffGNN6uVWlcjf5xlUEKy2o3g9czMTvwOYxJKMQrmqnSEM+tlxvtY	cancer registry	2025-06-18
NO_MATCH	1001000000080	F918273645	3SSmMfZ2osl9FmG9mXg4ZVorPNRzncM0rykfuDAKbHK6o0hZEyBdsuu/0ohaQvRa7v+hgkNfuwx5eMibbUG1GWgFMrvxDWl5OM0n	cancer registry	2025-06-18
NO_MATCH	1001000000097	V122143658	jL5spxyQuIMTYzn8Cbg4UHI0ZKrj3MnEkNroiJiTubvYDa631SIll8v/cnah090g9jKU00Gw94IqT/LLbIfGwHborX4HU4wU4IFN	cancer registry	2025-06-18
NO_MATCH	1001000000103	U823548996	folkP5a99415IRG4++libbLGLo46GsO4eAXXtaiw6Ef4OjahuwRYs+O/34ArJzYXbe2xCvDE0c18cOgjkhGkNRsXm7LGG8s8mCgG	cancer registry	2025-06-18
NO_MATCH	1001000000110	G472893579	wZ6UiSu3ihIXMwe+W5pjgYvGTY6pWzhO2TWTxN5hxG3EemfkKk5AmHPRtzPNOWzavcU3B9WXU+0OCA3z0/WM/oFOU8YrUxwyTIAT	cancer registry	2025-06-18
NO_MATCH	1001000000127	A565629888	5BI2OBbyUMbZNbpEPrq4QytGJy5rc8S5sxsDpau81rl5wdLI+/B954rVW78sOBxHXsKy42Wg5M7OzNrqRpGCMAjEOHemU8x0VEkc	cancer registry	2025-06-18
NO_MATCH	1001000000134	J182736459	9SSmUdR34ul/F2G9mDgcBXIrp9c3nclwvakfOTEefHq7ocxZkwBNlum/05heAPRa7vuBwkN3uwx7WcjQfCG/GWkFsjvQBWJ4dE0m	cancer registry	2025-06-18
NO_MATCH	1001000000141	W657483921	qjKnBCuQ63n7Zf8lnILYWCNUpu7rGii7cZRLnXTEyX/lOnSx1UFs7xseGnbk41tO9UTSQq2AUYRuZjfLYqrAJivCJN1s42ZcqF1K	cancer registry	2025-06-18
NO_MATCH	1001000000158	O584736259	nQ0eycUTGMMhdz3Ziyg8KqNjrH91tYC8vVpcOZGTcqht5b9gHfRdCsG3t8h+iv3A8BKpksXG46xri9qELaSOGnjkeN7gKiL4UOh1	cancer registry	2025-06-18

1-10 of 20 1 2

Download CSV

Figure 28 – Interface "Import" to download the CSV file

5. Exporting the PII and Bloom filters via export function

After importing PII (e.g. KVNR) as described in Chapter 4, you can retrieve the PII – Bloom filter assignment later using the E-PIX® export function.

Therefore, click in the E-PIX® menu under **"Lists"** on **"Export"** (see Figure 29).

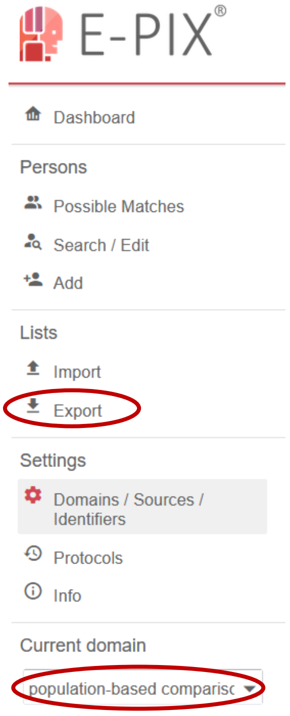

Figure 29 – E-PIX® menu

Note: If you are working in the demo system or have already created several domains, please make sure that the domain you need is selected under **"Current domain"** (see Figure 29).

Now, click on **“Export all persons”** (see Figure 30).

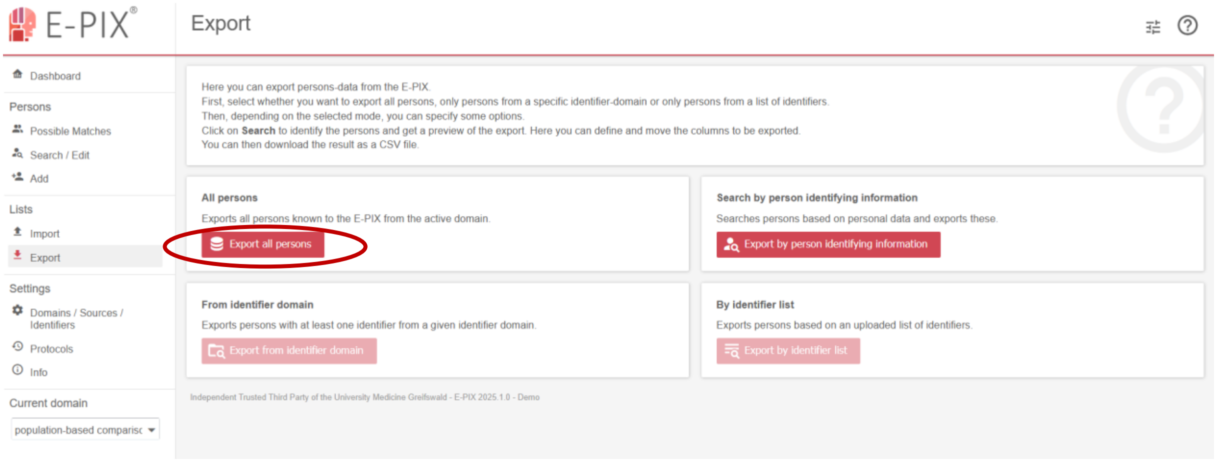

Figure 30 – Exporting data

Leave the check mark for the item **“Only export identifier value of the main identity”** and click on **“Search”** (see Figure 31).

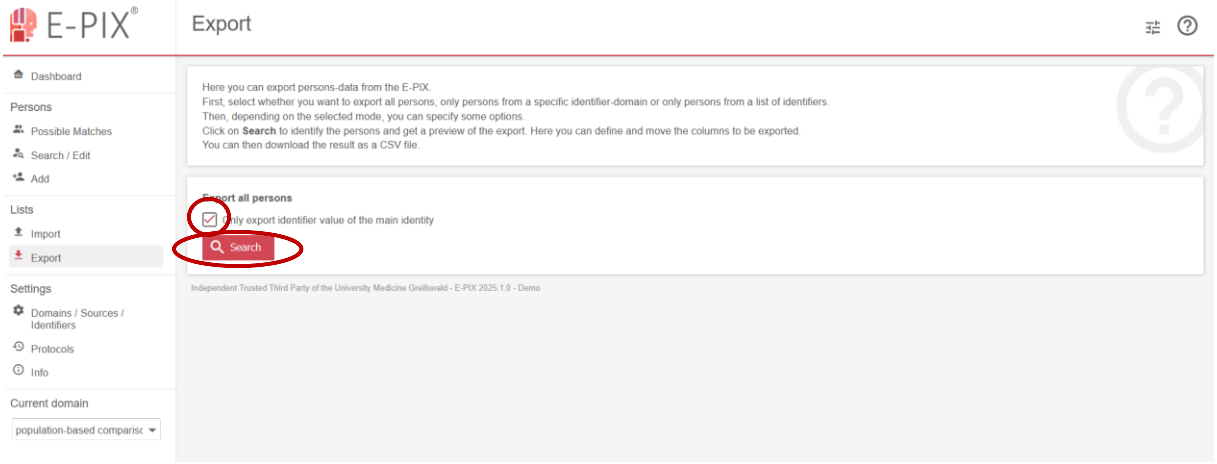

Figure 31 – Exporting data: Step "Export all persons"

Click on **“Exclude empty columns”** and then click on the cross in the column **“MPI”** to remove this variable form the result, too (see Figure 32).

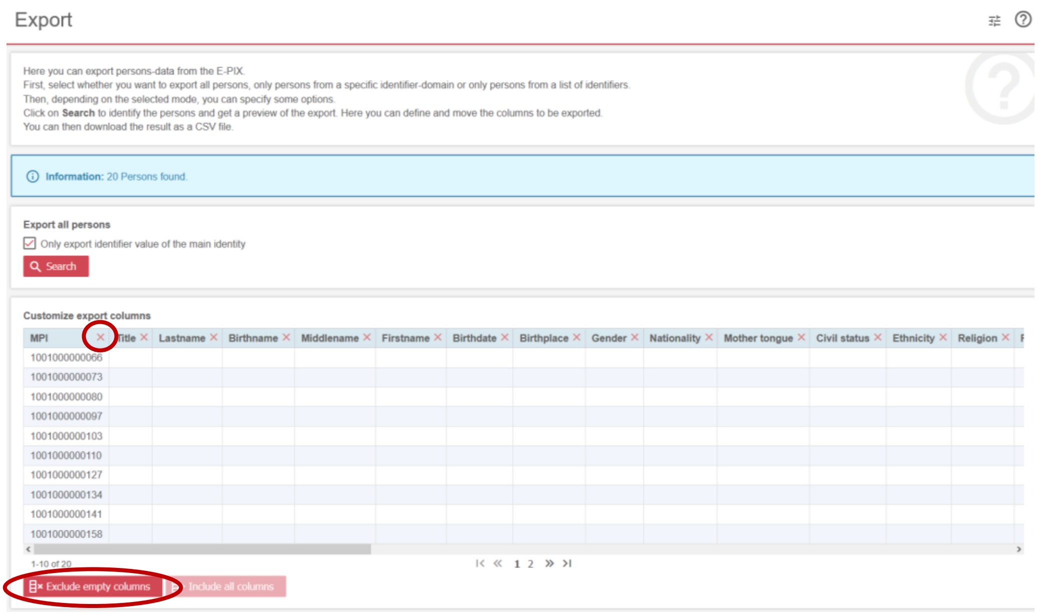

Figure 32 – Exporting data: Step “Customize export columns”

Note: The grayed-out columns are not exported. If you only want to export field 8 (Bloom filter) as a variable, you can also remove field 1 (KVNR) as a variable from the result by clicking on the cross in the **“Field 1”** column.

By using the horizontal scroll bar, you can view the two remaining variables, field 1 (KVNR) and field 8 (Bloom filter), which are exported in CSV format (see Figure 33[7]).

Click on **“Download CSV”** and the file will be saved in your download folder (see Figure 33).

[7] The health insurance numbers (KVNR) shown here are fictitious and do not correspond to real individuals.

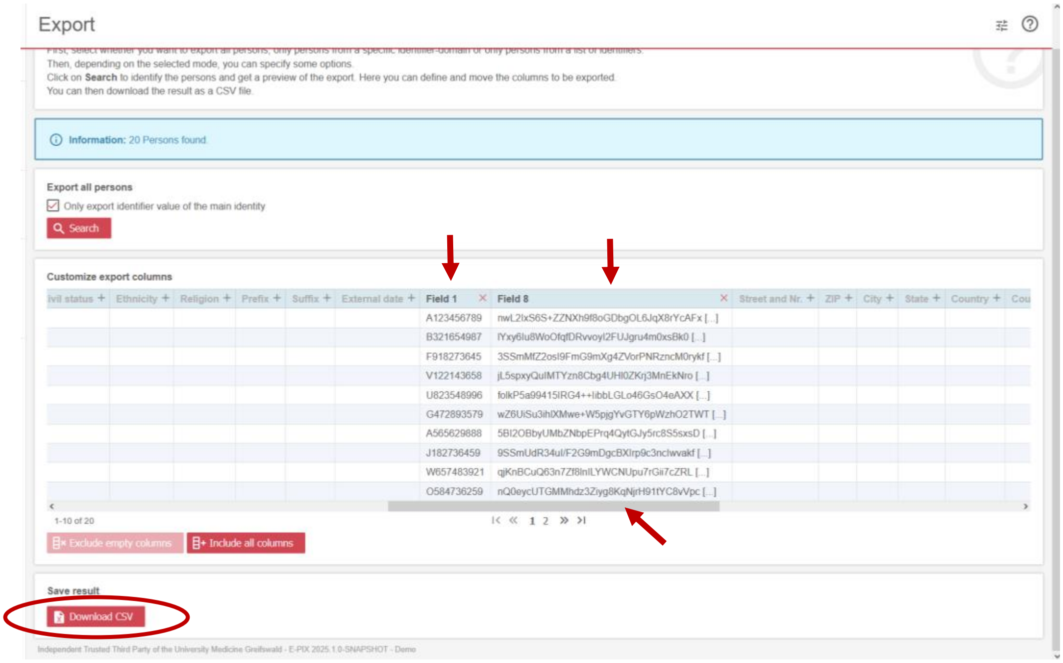

Figure 33 – Exporting data: Step "Save result"

Further information is provided in the manual in Chapter 8.6.

The THS software solutions such as the E-PIX® are increasingly being used in practice. The Independent Trusted Third Party of the University Medicine Greifswald has initiated the THS Community Dialog as a central point of contact for specific questions in order to promote the joint exchange between the project partners and users of the software solutions. Further information on the THS Community can be found under the following link:

https://www.ths-greifswald.de/community/

If you are interested, you can also register directly for the next THS Community Dialog via this page.
